# CD73-targeted CAR-NK cell therapy displays potent antitumor activity and translational potential in relapsed/refractory lung adenocarcinoma

**DOI:** 10.1186/s40364-026-01036-5

**Published:** 2026-10-28

**Authors:** Qianqian Liu, Qifei Dong, Yimei Chen, Nannan Wang, Dian Zhuang, Lihua Chen, Yanzhuo Ye, Shasha Huang, Wenzhao Zhong, Wei Kevin Zhang, Tao Xu, Jiaqi Sun, Huisheng Liu

**Affiliations:** 1 School of Biomedical Engineering, Guangzhou Medical University, Guangzhou, Guangdong 511495 China; 2 Guangzhou National Laboratory, Guangzhou, Guangdong 510320 China; 3 Institute of Cytology and Genetics, School of Basic Medical Sciences, Hengyang Medical School, University of South China, Hengyang, Hunan 421001 China; 4 Shantou University Medical College, Shantou, Guangdong 515041 China; 5 Guangdong Provincial People’s Hospital, Guangdong Lung Cancer Institute, Guangdong Academy of Medical Sciences, Southern Medical University, Guangzhou, Guangdong 510080 China; 6 Guangdong Provincial Key Laboratory of Translational Medicine in Lung Cancer, Guangzhou, Guangdong 510080 China; 7 School of Forestry and Biotechnology, Zhejiang A&F University, Hangzhou, China; 8 National Key Laboratory for Development and Utilization of Forest Food Resources, Zhejiang A&F University, Hangzhou, China

**Keywords:** CD73(NT5E), CAR-NK cell therapy, Relapsed/Refractory LUAD, Immune microenvironment remodeling

## Abstract

**Background:**

Relapsed/refractory (R/R) lung adenocarcinoma (LUAD) remains a clinical challenge with limited therapies and poor prognosis. CAR‑NK cell therapy has shown promise in solid tumors, but its efficacy is limited by both the availability of effective targets and the immunosuppressive tumor microenvironment. CD73 (NT5E), highly expressed across solid tumors, catalyzes adenosine production to drive immunosuppression, representing both a specific CAR‑NK target and a key leverage point to overcome tumor immune evasion. However, its systematic evaluation as a therapeutically actionable target for CAR‑NK cell therapy in R/R LUAD remains unexplored.

**Methods:**

We first characterized CD73 expression through integrative analysis of public datasets, primary and relapsed clinical LUAD specimens, and LUAD cell lines derived from primary and metastatic tumors. We then constructed CD73‑CAR‑NK cells and assessed their in vitro cytotoxicity across LUAD cell lines of varying malignancy, as well as their effects on extracellular adenosine and resistance to adenosine‑mediated immunosuppression. Finally, we assessed their antitumor efficacy in a stepwise manner across a tiered panel of in vivo models, including cell line‑derived xenograft (CDX), metastasis, patient‑derived xenograft (PDX), and clinically relevant immune‑humanized PDX models.

**Results:**

CD73 expression was elevated in metastasis-derived LUAD cell lines and R/R LUAD specimens. Accordingly, CD73-CAR-NK92 cells exhibited potent cytotoxicity against CD73-high LUAD cells, accompanied by an activated effector phenotype, reduced extracellular adenosine, partial resistance to adenosine-mediated suppression, and limited activity toward normal bronchial epithelial cells. Notably, this enhanced function was consistently observed across multiple NK cell sources including peripheral blood-derived NK (PBNK), umbilical cord blood‑derived NK (UBNK), and iPSC-derived NK (iNK). In vivo, CD73‑CAR‑NK92 cells significantly suppressed tumor growth and metastasis, with enhanced tumor‑site enrichment, prolonged retention, and elevated intratumoral levels of TNF‑α and IFN‑γ. Importantly, in an immune‑humanized PDX mouse model, CD73‑CAR‑NK92 cells significantly reduced the growth of R/R patient‑derived xenografts, accompanied by immune remodeling, without marked systemic inflammation during the observation period.

**Conclusions:**

These findings provide preclinical evidence that CD73-CAR-NK cells exhibit potent antitumor efficacy for R/R LUAD through their dual functions of eliminating CD73-high tumor cells and resisting adenosine-mediated immunosuppressive signaling, supporting their therapeutic potential.

**Supplementary Information:**

The online version contains supplementary material available at https://doi.org/10.1186/s40364-026-01036-5.

## Introduction

Lung adenocarcinoma (LUAD) remains a leading cause of cancer-related mortality worldwide, with a substantial proportion of patients experiencing tumor recurrence and metastasis after initial treatment due to immune evasion and therapeutic resistance [1–4]. For relapsed/refractory (R/R) LUAD, identifying specific targets for effective CAR-based cell therapy remains a critical clinical challenge. Although CAR-based immunotherapies have achieved remarkable success in hematological malignancies, their efficacy against solid tumors has been limited largely by a scarcity of tumor-specific targets [5–7]. Even in trials showing promising signals, durable responses remain restricted to patients with high and homogenous antigen expression, and CAR-T and CAR-NK trials in lung cancer have been constrained by on-target/off-tumor toxicity or limited efficacy due to the immunosuppressive microenvironment [5–8].

CD73 (NT5E), the rate‑limiting enzyme of extracellular adenosine production, is abundantly overexpressed across multiple solid tumors and strongly associated with poor prognosis [9–11]. CD73 drives immune evasion through a dual mechanism: adenosine‑mediated suppression of effector T cells and NK cells, and non‑enzymatic promotion of epithelial‑to‑mesenchymal transition (EMT) and tumor metastasis [11–18]. Among all solid tumors, non‑small cell lung cancer shows one of the highest levels of CD73 expression, further amplified by oncogenic drivers such as mutant EGFR and KRAS [19–22]. Although monoclonal antibodies and small‑molecule inhibitors targeting CD73 are under clinical evaluation, they lack direct tumoricidal activity and cannot counteract the non‑enzymatic functions of CD73 [23–27]. CAR‑NK cell therapy offers a compelling alternative by combining antigen‑specific targeting with intrinsic cytotoxicity and a favorable safety profile [28–32]. However, adenosine directly impairs NK cell function via A2A receptor signaling [33–37]. Targeting CD73 with a CAR‑NK strategy may provide a dual therapeutic benefit by directly killing CD73⁺ tumor cells while alleviating adenosine-mediated immunosuppression.

In this study, we systematically evaluated CD73-targeted CAR-NK cells against R/R LUAD through in vitro and in vivo approaches. CD73 was highly expressed in R/R LUAD specimens, and CD73-CAR-NK cells showed potent antigen-dependent cytotoxicity and significant tumor suppression, accompanied by increased immune-cell infiltration and preliminary evidence of short-term tolerability in clinically relevant immune-humanized PDX models. These findings provide a preclinical rationale for further investigation of CD73-targeted CAR-NK cell therapy in R/R LUAD.

## Materials and methods

For details on the information presented this section, please also see the supplementary figures and tables.

### Ethics approval and consent to participate

The tissue collection procedure was conducted in strict accordance with the guidelines reviewed and approved by the Ethics Committee of Guangdong Provincial People’s Hospital (Approval ID: KY-Q-2021-019-12). All donors signed informed consent forms for scientific research. All animal experimental procedures were performed in compliance with the specifications formulated by the Institutional Animal Care and Use Committee of Guangzhou Laboratory. All experiments in this study have been approved by the Ethics Committee of Guangzhou Laboratory (Approval ID: GZLAB-AUCP- 2024-03-A05).

### Lentivirus Production and Generation of CAR- NK cells

The second-generation CAR plasmid targeting CD73 (containing anti-CD73 scFv, 4-1BB, and CD3ζ) was constructed and synthesized by Alberson Biology Company. Lentivirus was produced by co-transfecting HEK293T cells with this plasmid and the packaging plasmids (psPAX2, pMD2.G). Supernatants were harvested, filtered, and concentrated at 48-72 h post-transfection. NK92 cells were transduced with the concentrated virus. At 48 h post-transduction, puromycin was added to the culture medium at a final concentration of 2.5 µg/mL to select stably transduced cells. CD73-CAR expression was confirmed via ZsGreen1 reporter, and CD73-CAR-positive NK92 cells were expanded for subsequent functional assays. Rigorous quality control was implemented during the preparation of CD73-CAR-NK92 cells.

### Flow cytometry

Cells were collected and stained with fluorochrome-conjugated antibodies at a 1:500 dilution for 30 min on ice in the dark. Data were acquired using an Agilent Novocyte Advanteon flow cytometer and analyzed with FlowJo software (version 10.10). Detailed antibody information is provided in Supplementary Table 4.

### Western blot analysis

Total proteins were extracted using RIPA lysis buffer supplemented with protease and phosphatase inhibitors. For PKA substrate phosphorylation analysis, cells in the H1299 co-culture group were incubated with H1299 cells at an E: T ratio of 4:1 for 6 h before protein collection. For the adenosine stimulation group, cells were treated with 10 µM adenosine for 30 min immediately before harvesting. Protein concentrations were determined by BCA assay, and 30 µg of denatured protein per sample was separated by SDS-PAGE and transferred to PVDF membranes. After blocking, membranes were incubated overnight at 4 °C with primary antibodies against CD73 (1:1000, Abcam), phospho-PKA substrate (1:1000, CST), and GAPDH (1:5000, CST) as a loading control, followed by HRP-conjugated secondary antibodies. Protein bands were visualized by ECL and quantified using ImageJ (version 2.9.0). CD73 expression was calculated as the ratio of CD73 to GAPDH, and PKA activation was assessed as the ratio of phosphorylated PKA substrate to GAPDH. All experiments were performed in triplicate.

### In vitro cytotoxicity assay

For 2D cytotoxicity assays, H1299-td-luc, PC-9-td-luc, or A549-EGFP cells were co-cultured with effector cells at effector-to-target (E: T) ratios of 0.5:1, 1:1, 2:1, 4:1, or 8:1, and images were acquired every 1 h for 24 h. For 3D spheroid assays, the same tumor cell lines were seeded into ultra-low attachment plates 48 h before the addition of effector cells to allow spheroid formation. Effector cells were then added at the indicated E: T ratios, and images were acquired every 2 h for 48 h. All live-cell imaging was performed using the IncuCyte^®^ S3 Live-Cell Analysis System (Sartorius), and images were processed with ImageJ software.

### Xenograft mouse models

All animal experiments were approved by and performed in accordance with the guidelines of the Animal Care and Use Committee of Guangzhou Laboratory. Mice were obtained from Guangdong Yaokang Biotechnology Co., Ltd.

For subcutaneous xenograft models, 6 × 10⁶ H1299-td-luc or PC9-td-luc cells were injected subcutaneously into 6-8-week-old NCG mice. For the lung metastasis model, 3 × 10⁶ H1299-td-luc cells were injected intravenously via the tail vein into 6-8-week-old female NCG mice.

For patient-derived xenograft (PDX) models, fresh tumor fragments (~ 50 mm³) were implanted subcutaneously into 6-8-week-old NSG mice or 20-21-week-old huHSC-NCG mice. Because of limited specimen availability, tumor tissue from each patient was divided as evenly as possible among the PBS, NK92, and CD73-CAR-NK92 groups to maintain a patient-matched design; conventional randomization was not used. The huHSC-NCG mice were obtained from Guangdong Yaokang Biotech as a single batch and generated by transplantation of human CD34⁺ hematopoietic stem cells into sublethally irradiated NCG mice. According to supplier quality-control data, human hematopoietic reconstitution was established before use.

Tumor engraftment was confirmed before treatment by bioluminescence imaging or caliper measurement. Treatment allocation and outcome assessment were not blinded, and tumor measurements were performed according to pre-specified standardized procedures.

### Statistical analysis

Statistical analyses were performed using GraphPad Prism (version 10.1.2). Data are presented as mean ± SD. Normality was assessed using the Shapiro-Wilk test. Two-group comparisons were performed using an unpaired two-tailed Student’s *t*-test or Mann-Whitney *U* test, as appropriate. Multiple independent groups were analyzed by one-way ANOVA with Tukey’s or Dunnett’s multiple-comparison test. Matched CD73 IHC data from normal, adjacent, and tumor tissues obtained from the same patients were analyzed using a repeated-measures mixed-effects model. Longitudinal tumor-volume and bioluminescence data were analyzed using two-way repeated-measures ANOVA or mixed-effects models with correction for multiple comparisons. Real-time cytotoxicity data were analyzed by comparing area-under-the-curve (AUC) values using one-way ANOVA with Tukey’s test. Survival was assessed by Kaplan-Meier analysis and the log-rank test. RNA-seq differential expression was analyzed using DESeq2 with Benjamini-Hochberg FDR correction; DEGs were defined as |log2 fold change| ≥ 1 and adjusted *P* < 0.05. A two-sided *P* < 0.05 was considered statistically significant.

## Results

### CD73 is elevated in relapsed/refractory LUAD and associated with poor prognosis

**Fig. 1:**
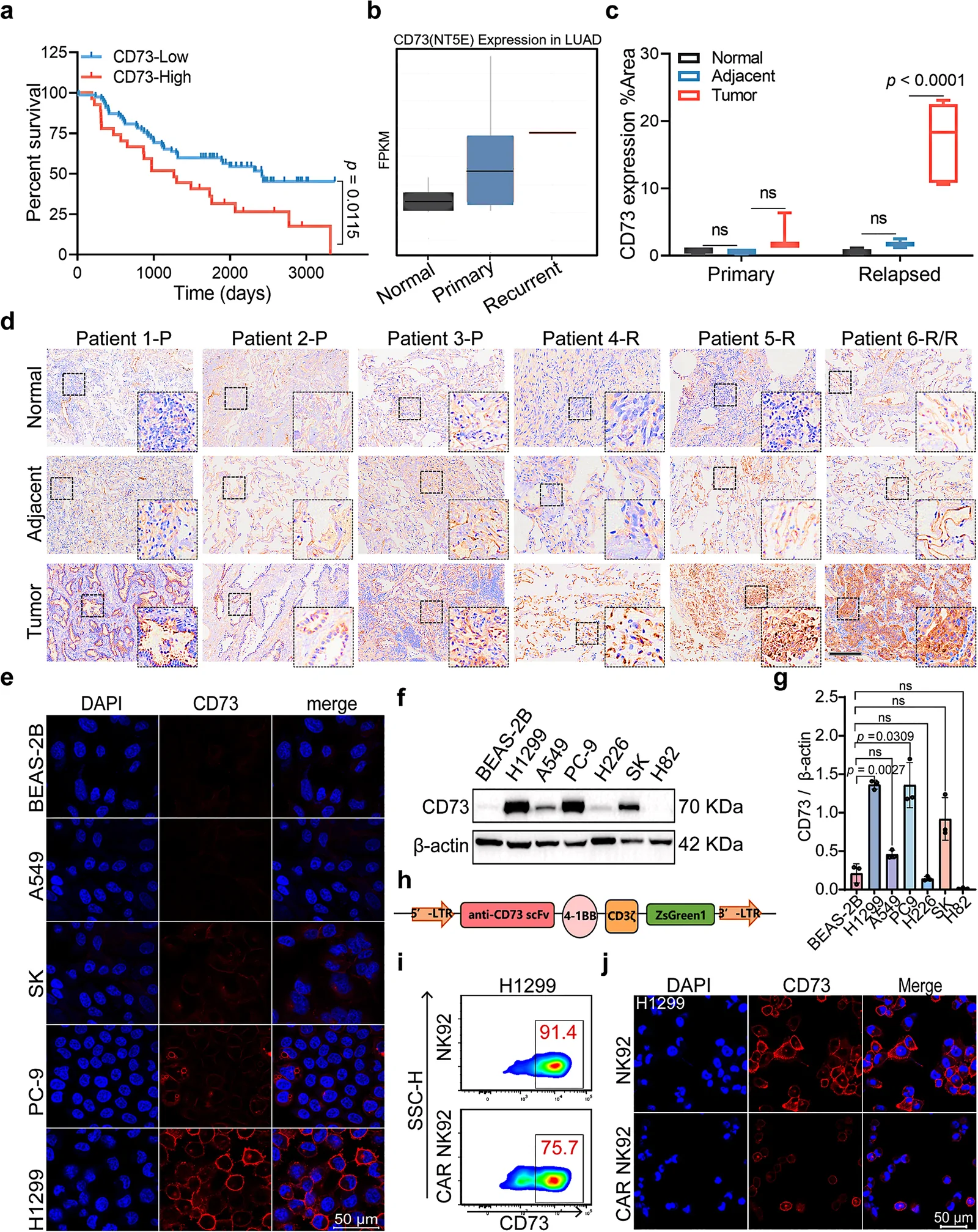
CD73 is upregulated in relapsed/refractory LUAD and serves as a rational target for CAR NK therapy. **(a)** Kaplan-Meier analysis of overall survival in LUAD patients stratified by CD73 expression using data from The Human Protein Atlas (HPA) database (CD73-Low, *n* = 78; CD73-High, *n* = 28). Statistical significance was determined by the log-rank test. **(b) ***NT5E (CD73)* mRNA expression in normal lung tissues (*n* = 58), primary LUAD tumors (*n* = 528), and recurrent LUAD tumors (*n* = 2) from the GDC TCGA Lung Adenocarcinoma (LUAD) cohort accessed through the UCSC Xena platform. Gene expression was presented as FPKM-normalized RNA-sequencing data. The recurrent subgroup is presented descriptively because of the limited number of available recurrent tumor samples. **(c**,** d)** Quantification of CD73 immunohistochemical (IHC) staining intensity (**c**) and representative IHC images (**d**) of CD73 protein expression in primary LUAD tissues (*n* = 3) and R/R LUAD tissues (*n* = 6), as well as normal lung and adjacent normal tissues. Scale bar in (**d**), 100 μm. **(e)** Immunofluorescence staining of CD73 (red) in A549, SK, PC9, H1299, and BEAS-2B cell lines. Nuclei were counterstained with DAPI (blue). Scale bar, 50 μm. **(f**,** g)** Western blot analysis (**f**) and corresponding densitometric quantification (**g**) of CD73 protein expression in LUAD cell lines and BEAS-2B cells. β-actin served as a loading control. Quantification was performed with ImageJ. **(h)** Schematic representation of the second-generation CD73-specific CAR construct, comprising an anti-CD73 single-chain variable fragment (scFv), 4-1BB costimulatory domain, and CD3ζ signaling domain. **(i)** Flow cytometry of surface CD73 on H1299 cells after 4 h co-culture with NK92 or CD73-CAR-NK92 cells. Reduced signal indicates CAR-mediated antigen engagement. **(j)** Immunofluorescence images of H1299 cells stained for surface CD73 (red) after 4 h co-culture with NK92 or CD73-CAR-NK92 cells. Scale bar, 50 μm. Data in **(c**,** g)** are presented as mean ± SD. Statistical significance in (**a**) was determined by the log-rank test; in (**c**) by a mixed-effects model with repeated measures accounting for within-subject matching of tissue types, followed by Tukey’s multiple comparisons test; and in (**g**) by one-way ANOVA followed by Dunnett’s multiple comparisons test, using BEAS-2B as the reference control group

We first assessed the clinical relevance of CD73 in LUAD by examining its association with patient outcomes and disease status. Kaplan-Meier analysis showed that patients with high CD73 expression had significantly worse overall survival than those with low CD73 expression (Fig. 1a), indicating an association between elevated CD73 expression and unfavorable clinical outcome. Exploratory analysis of the GDC TCGA Lung Adenocarcinoma (LUAD) cohort showed higher *NT5E* (CD73) mRNA expression in the two recurrent LUAD samples relative to the expression distributions observed in normal lung and primary LUAD tissues (Fig. 1b). However, because only two recurrent tumor samples were available in this cohort, this observation was considered descriptive rather than evidence of biomarker validation. Immunohistochemical analysis of our independent clinical cohort (primary LUAD, *n* = 3; R/R LUAD, *n* = 6) further showed higher CD73 protein expression in R/R LUAD tumors than in primary tumors (Fig. 1c, d). Although the limited clinical sample size precludes definitive biomarker validation, these complementary findings provide clinical rationale for further evaluating CD73 as a clinically relevant therapeutic target in R/R LUAD.

Prompted by this enrichment, we profiled CD73 expression across a panel of LUAD cell lines. Consistently, immunofluorescence showed prominent membrane‑localized CD73 staining in A549 and SK cells, whereas the lymph node metastasis‑derived PC-9 and H1299 cells exhibited the highest CD73 expression, and CD73 was virtually undetectable in normal bronchial epithelial BEAS‑2B cells (Fig. 1e). Western blot analysis revealed abundant CD73 protein in LUAD cells yet only negligible levels in normal epithelial cells (Fig. 1f, g), supporting CD73 as an accessible surface target.

Accordingly, we constructed a second‑generation CD73‑targeting CAR composed of an anti‑CD73 scFv linked to 4‑1BB and CD3ζ intracellular signaling domains (Fig. 1h, Supplementary Fig. 1a). Bright-field and fluorescence imaging confirmed successful expansion of ZsGreen-positive CD73-CAR-NK92 cells, and flow cytometry at 48 h post-transduction showed an initial transduction efficiency of approximately 50% before antibiotic selection (Fig. 1i, j; Supplementary Fig. 1b, c). CAR-transduced cells were expanded for subsequent functional assays. Together, these findings support CD73 as a clinically relevant surface target for further evaluation in R/R LUAD and establish the CD73-CAR-NK92 platform used in subsequent functional studies.

### CD73-CAR-NK92 cells exhibit an activated effector phenotype and enhanced cytotoxic responses

To characterize the transcriptional changes associated with CD73-CAR engineering, we performed bulk RNA sequencing of parental NK92 and CD73‑CAR‑NK92 cells. Differentially expressed genes (DEGs) were defined as those with |log₂ fold change| ≥ 1 and adjusted *P* < 0.05. KEGG pathway enrichment analysis showed enrichment of DEGs in pathways related to protein processing in the endoplasmic reticulum, cytokine-cytokine receptor interaction, oxidative phosphorylation, TNF signaling, p53 signaling, antigen processing and presentation, and ferroptosis (Supplementary Fig. 2a). These pathway‑level changes suggest that CD73-CAR engineering was associated with transcriptional changes involving cytokine signaling, cellular stress responses, metabolism, and immune effector function.

**Fig. 2:**
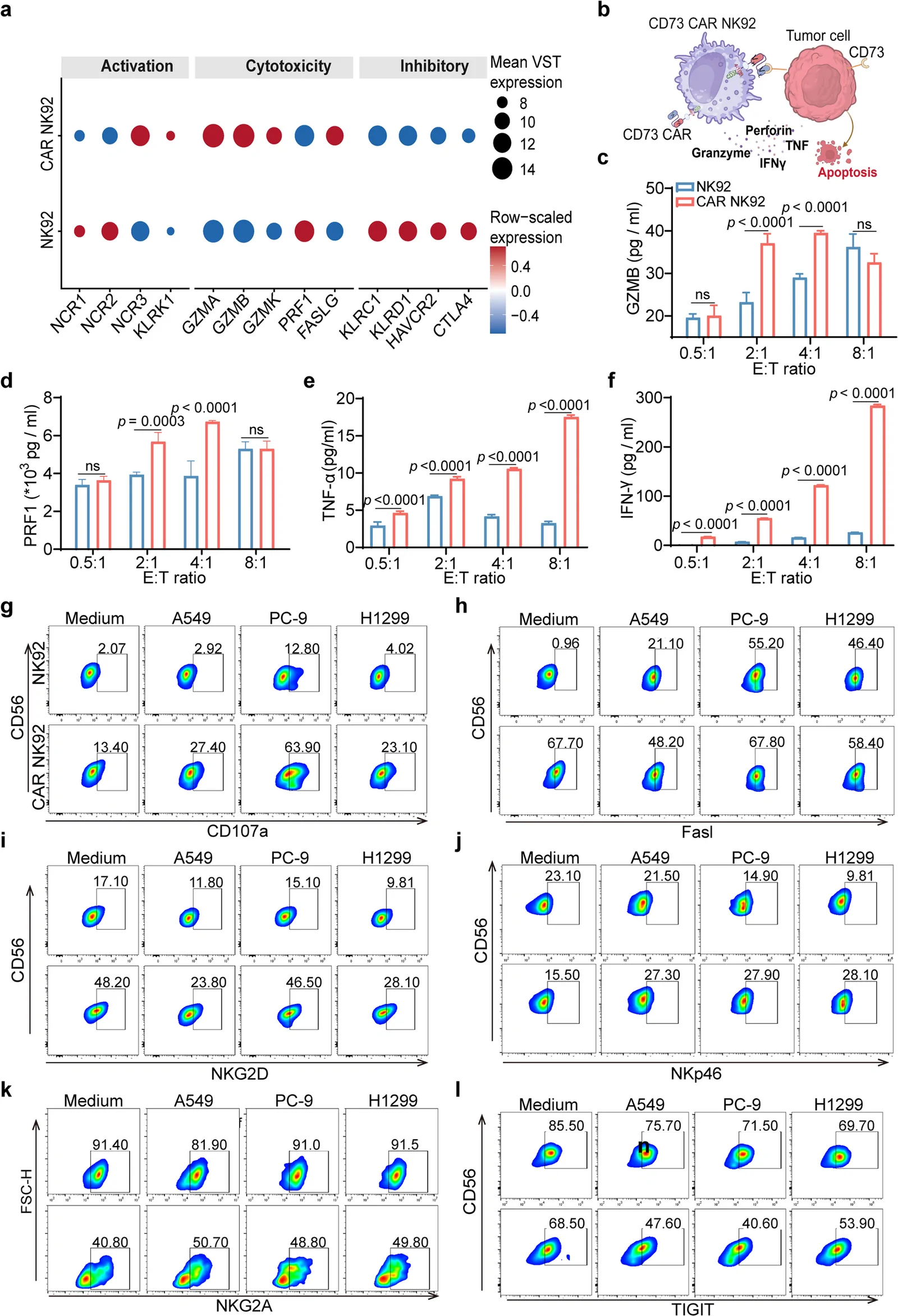
CD73-CAR-NK92 cells exhibit enhanced effector function and an activated phenotype. **(a)** Bubble plot showing differentially expressed genes related to activation markers, cytotoxic effectors, and inhibitory markers between CD73‑CAR‑NK92 and NK92 cells, as identified by bulk RNA sequencing. Bubble size represents the absolute fold change, and color intensity indicates statistical significance. **(b)** Schematic illustration depicting the recognition and elimination of CD73⁺ tumor cells by CD73‑CAR‑NK92 cells. **(c-f)** ELISA quantification of GZMB **(c)**, PRF1 (**d**), TNF‑α **(e)**, and IFN‑γ **(f)** in co‑culture supernatants. **(g-j)** Representative FlowJo‑generated pseudocolor density plots showing expression of CD107a **(g)**, FASL **(h)**, NKG2D **(i)**, and NKp46 **(j)** on NK92 cells (top row) and CD73‑CAR‑NK92 cells (bottom row) following tumor co‑culture. **(k**,** l)** Representative pseudocolor density plots of TIGIT **(k)** and NKG2A **(l)** expression. For all flow cytometry panels, blue‑to‑red pseudocolor gradients represent increasing cell density. Data in **(c-f)** are presented as mean ± SD from three independent experiments. Statistical significance was determined using a two-way ANOVA followed by Šídák’s multiple comparisons test (or a Mixed-effects model if the data show unequal variances)

We next examined representative genes related to NK-cell activation, cytotoxicity, migration, cytokine/chemokine signaling, and receptor expression. CD73‑CAR‑NK92 cells displayed increased expression of selected cytokine and chemokine‑associated genes, as well as migration‑related receptors (Fig. 2a, Supplementary Fig. 2b). Several cytotoxicity‑associated genes, including *FASLG*, *granzyme A (GZMA)*, and *granzyme B (GZMB)*, also showed higher expression in CD73‑CAR‑NK92 cells, whereas *KLRC1*, *KLRD1*, and *HAVCR2*, were reduced (Fig. 2a, Supplementary Fig. 2b). Notably, these changes were selective rather than uniformly distributed across all effector or inhibitory markers, indicating that CD73‑CAR engineering induces a targeted transcriptional remodeling of NK92 cells rather than a global activation program.

Prompted by these transcriptional findings, we next evaluated the functional phenotype of CD73‑CAR‑NK92 cells upon tumor encounter. CAR‑engineered NK cells execute a coordinated cytotoxic program encompassing target cell engagement, degranulation, perforin (PRF1) and GZMB release, proinflammatory cytokine secretion, and subsequent tumor cell apoptosis [30, 38, 39]. To evaluate whether CD73 targeting amplifies NK cell responsiveness against LUAD, we co‑cultured NK92 or CD73‑CAR‑NK92 cells with A549, PC‑9, and H1299 cell lines, and systematically assessed functional mediator release and surface receptor expression (Fig. 2b). Following co‑culture with CD73‑high H1299 cells, ELISA analysis revealed that PRF1, GZMB, TNF‑α, and IFN‑γ levels were prominently elevated in the CD73‑CAR‑NK92 group relative to the NK92 group (Fig. 2c-f), indicating augmented granule‑mediated cytotoxicity and strengthened proinflammatory activity. At the phenotypic level, co‑culture with LUAD cell lines significantly upregulated activation markers CD107a, FASL, NKG2D, and NKp46 on CD73‑CAR‑NK92 cells (Fig. 2g-j; Supplementary Fig. 2c-f). Elevated CD107a indicated enhanced degranulation, while concurrent increases in FASL, NKG2D, and NKp46 suggested reinforced tumor recognition and killing. In parallel, inhibitory receptors TIGIT and NKG2A were significantly reduced in CD73‑CAR‑NK92 cells (Fig. 2k, l; Supplementary Fig. 2g, h), suggesting that CAR engagement may relieve tumor-induced inhibitory signaling.

Collectively, these findings show that CD73-CAR-NK92 cells exhibit selective transcriptional changes together with an enhanced effector phenotype characterized by increased cytotoxic mediator release, higher expression of activation-associated molecules, and reduced expression of selected inhibitory receptors. These functional changes support the enhanced antitumor activity of CD73-CAR-NK92 cells observed in subsequent experiments.

### CD73-CAR-NK cells exhibit target-specific cytotoxicity and partial resistance to adenosine-mediated suppression

**Fig. 3:**
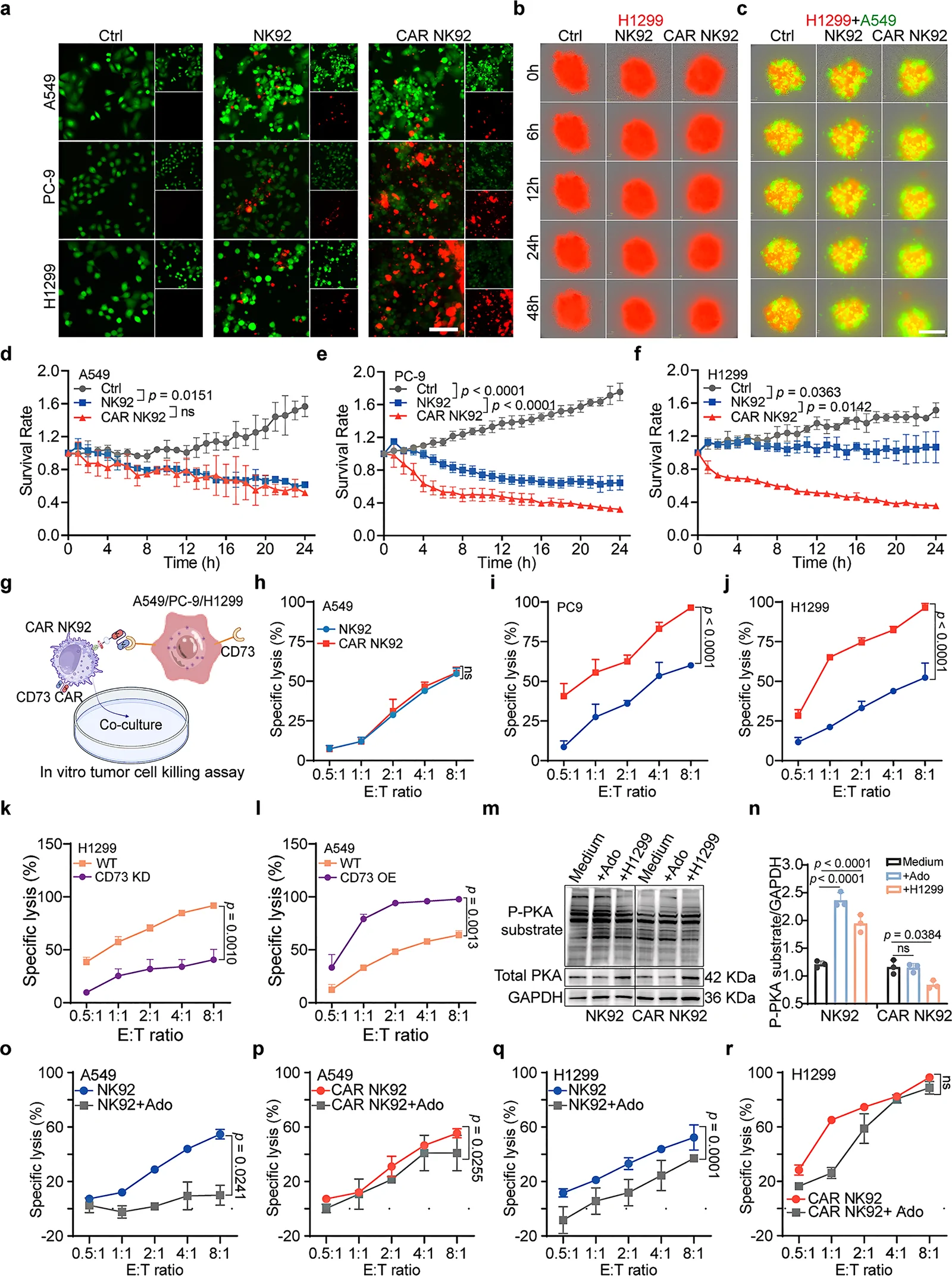
CD73-CAR-NK cells mediate antigen-dependent cytotoxicity and resist adenosine-mediated suppression. **(a)** Live/dead staining showing specific cytotoxicity of NK92 and CD73‑CAR‑NK92 cells against A549 (CD73-low), PC-9 (CD73-medium), and H1299 (CD73-high) cells at an effector‑to‑target ratio of 4:1. Scale bar, 100 μm. **(b)** Representative bright‑field images of H1299 tumor spheroids treated with PBS, NK92 cells, or CD73‑CAR‑NK92 cells. Scale bar, 400 μm. **(c)** Representative images of mixed H1299/A549 spheroids showing preferential elimination of CD73⁺ H1299 cells (red) by CD73‑CAR‑NK92 cells. Scale bar, 400 μm. **(d-f)** Real‑time viability curves of A549 **(d)**, PC-9 **(e)**, and H1299 **(f)** cells co‑cultured with NK92 or CD73‑CAR‑NK92 cells over 24 h, expressed as percentage of viable tumor cells relative to time zero. **(g)** Schematic of the co‑culture killing assay. **(h-j)** Cytotoxicity of NK92 and CD73‑CAR‑NK92 cells against A549 **(h)**, PC9 **(i)**, and H1299 **(j)** cells at the indicated effector‑to‑target (E: T) ratios. **(k)** Cytotoxicity of CD73-CAR-NK92 cells against control (si-NC) and CD73-knockdown (CD73-KD) H1299 cells. **(l)** Cytotoxicity of CD73-CAR-NK92 cells against vector control and CD73-overexpressing (CD73-OE) A549 cells. **(m)** Western blot analysis of phosphorylated PKA substrate levels in NK92, and CD73-CAR-NK92 cells under the indicated conditions (untreated, exogenous adenosine, or H1299 co-culture). **(n)** Densitometric quantification of phosphorylated PKA substrate levels normalized to the loading control. (**o**,** p**) Cytotoxicity of NK92 cells **(o)** and CD73-CAR-NK92 cells **(p)** against A549 cells in the presence or absence of exogenous adenosine (10 µM) at the indicated E: T ratios. (**q**,** r**) Cytotoxicity of NK92 cells **(q)** and CD73-CAR-NK92 cells **(r)** against H1299 cells in the presence or absence of exogenous adenosine (10 µM) at the indicated E: T ratios. Images in **(a-c)** and curves in (**d–f**) are representative of three independent experiments. Data in (**h-l**,** n**,** o-r**) are presented as mean ± SD (*n* = 3). For (**d-f**), the area under the curve (AUC) was calculated for each viability curve, and differences in AUC among the indicated experimental groups were assessed by one-way ANOVA followed by Tukey’s multiple comparisons test. Other statistical comparisons were performed using unpaired two-tailed Student’s t-test for two-group comparisons or one-way ANOVA with Tukey’s post hoc test for multi-group comparisons where applicable. Statistical significance was set at *P* < 0.05

We next assessed the cytotoxic activity and target specificity of CD73-CAR-NK92 cells using A549 (CD73-low), PC-9 (CD73-medium), and H1299 (CD73-high) cells. CD73-CAR-NK92 cells showed only modest cytotoxicity against CD73-low A549 cells, comparable to NK92 cells, but significantly enhanced killing of CD73-high PC-9 and H1299 cells, with the strongest effect observed against CD73-high H1299 cells (Fig. 3a). These findings showed that the magnitude of CD73-CAR-NK92-mediated cytotoxicity was associated with target-cell CD73 expression. Neither NK92 nor CD73-CAR-NK92 cells induced appreciable cytotoxicity against normal BEAS-2B bronchial epithelial cells, with killing remaining below 20% across all E: T ratios tested (Supplementary Fig. 3a), supporting selective sparing of CD73-low normal cells in this setting.

Three-dimensional spheroid models further supported the enhanced antitumor activity of CD73-CAR-NK92 cells in a more physiologically relevant 3D setting. CD73-CAR-NK92 cells induced marked shrinkage of H1299 spheroids and near-complete elimination of the CD73-high H1299 population in H1299/A549 mixed spheroids (Fig. 3b, c). Similar results were obtained with PC-9 and PC-9/A549 mixed spheroids (Supplementary Fig. 3b, c). Real-time imaging showed that NK92 and CD73-CAR-NK92 cells suppressed A549 cells comparably, whereas CD73-CAR-NK92 cells more rapidly reduced PC-9 and H1299 tumor-cell burden (Fig. 3d-f). Across the tested E: T ratios, both groups showed similar cytotoxicity against A549 cells, whereas CD73-CAR-NK92 cells achieved near-complete killing of PC-9 and H1299 cells compared with approximately 50-60% killing by parental NK92 cells (Fig. 3g-j).

We further confirmed the antigen dependence of CD73-CAR-NK92 cytotoxicity by genetic manipulation of CD73 expression. siRNA-mediated CD73 knockdown in H1299 cells attenuated the cytotoxic advantage of CD73-CAR-NK92 cells, whereas enforced CD73 overexpression in A549 cells enhanced CAR-mediated killing (Fig. 3k, l; Supplementary Fig. 3e-i).

Moreover, following co-culture with H1299 cells, CD73-CAR-NK92 cells were associated with lower extracellular adenosine levels than NK92 cells (Supplementary Fig. 3d), likely reflecting CAR-mediated elimination of CD73-high tumor cells, a major source of adenosine. To examine the effect of CAR engineering on adenosine-mediated inhibitory signaling, we assessed PKA substrate phosphorylation as a downstream readout of A2AR-cAMP-PKA signaling. Exogenous adenosine and H1299 co-culture increased PKA substrate phosphorylation in NK92 and PBNK cells, whereas no significant increase was observed in CD73-CAR-NK92 or CD73-CAR-PBNK cells (Fig. 3m, n; Supplementary Fig. 3j, k). Consistent with these signaling changes, exogenous adenosine markedly impaired NK92 cytotoxicity, whereas CD73-CAR-NK92 cells largely retained killing activity across the tested E: T ratios (Fig. 3o-r). A2AR blockade partially restored NK92 cytotoxicity in the presence of adenosine, and anti-CD73 antibody treatment likewise enhanced NK92 killing (Supplementary Fig. 3l, m). These findings indicate that CD73-CAR-NK cells are less susceptible to adenosine-mediated inhibition.

Together, these data suggest that CD73-CAR-NK92 cells may lower extracellular adenosine primarily by eliminating CD73-high tumor cells while remaining less susceptible to exogenous adenosine, thereby preserving cytotoxic function under adenosine-rich conditions.

### CD73-CAR modification confers consistent cytotoxicity across multiple NK cell sources

**Fig. 4:**
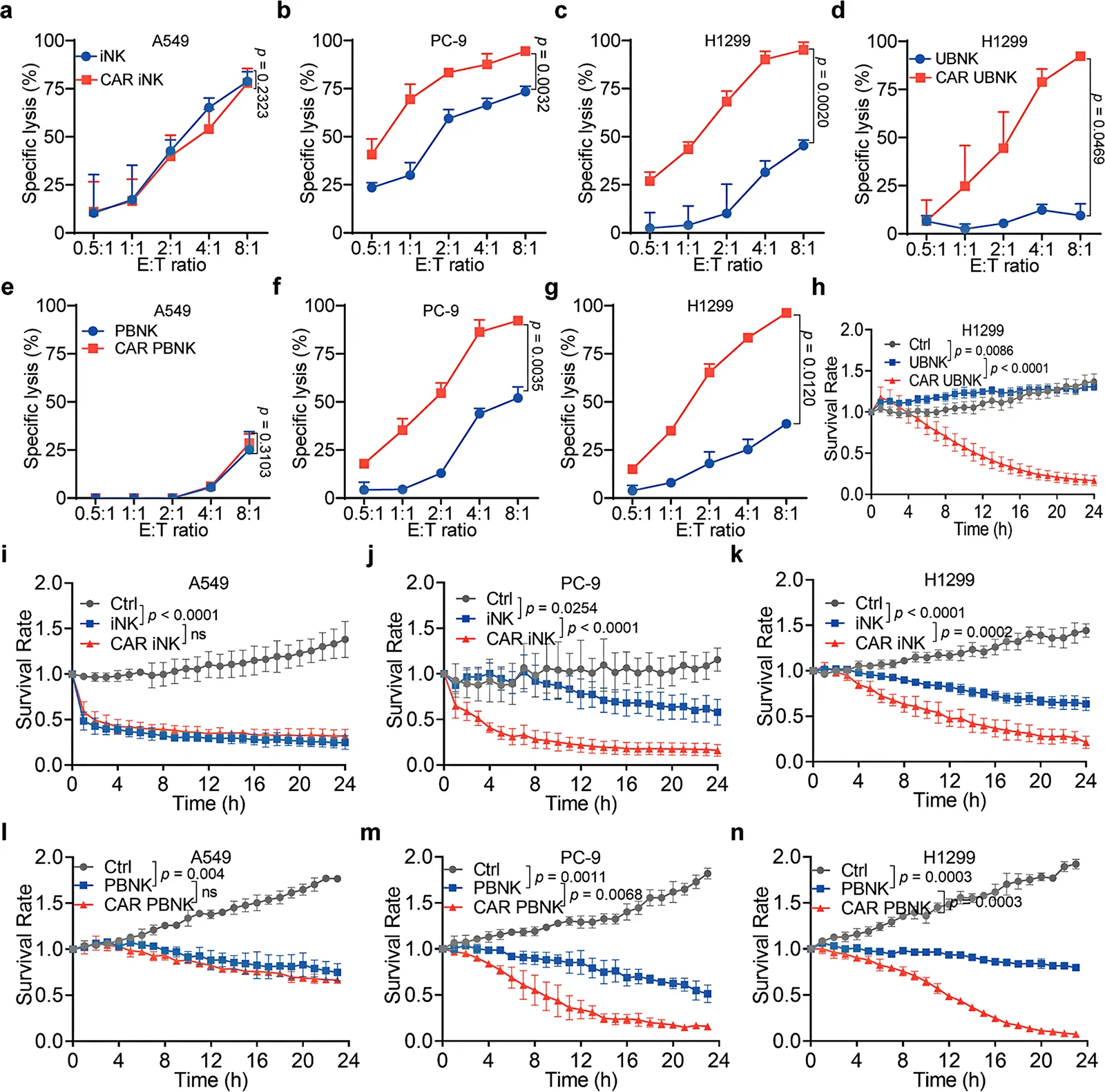
CD73-CAR function in NK cells from diverse sources. **(a-c)** Cytotoxicity of iNK and CD73-CAR-iNK cells against A549 **(a)**, PC9 **(b)**, and H1299 **(c)** cells at the indicated E: T ratios. **(d)** Cytotoxicity of UBNK and CD73-CAR-UBNK cells against H1299 cells at the indicated E: T ratios. **(e-g)** Cytotoxicity of PBNK and CD73-CAR-PBNK cells against A549 (**e**), PC9 **(f)**, and H1299 **(g)** cells at the indicated E: T ratios. **(h)** Real-time viability curve of H1299 cells co-cultured with UBNK or CD73-CAR-UBNK cells at an 8:1 E: T ratio over 24 h, expressed as percentage of viable tumor cells. **(i-k)** Real-time viability curves of A549 **(i)**, PC9 **(j)**, and H1299 **(k)** cells co-cultured with iNK or CD73-CAR-iNK cells at an 8:1 E: T ratio over 24 h. **(l-n)** Real-time viability curves of A549 **(l)**, PC9 **(m)**, and H1299 **(n)** cells co-cultured with PBNK or CD73-CAR-PBNK cells at an 8:1 E: T ratio over 24 h. Data are presented as mean ± SD unless otherwise indicated. For (**a-g**), a two-way ANOVA mixed-effects model was used, and P values were derived from the column factor. For (**h-n**), the area under the curve (AUC) was calculated for each biological replicate, and differences in AUC among the indicated groups were assessed by one-way ANOVA followed by Tukey’s multiple comparisons test

We next examined whether the enhanced antitumor activity of CD73-CAR-NK92 cells could be reproduced across alternative NK-cell sources, an important consideration for the development of allogeneic “off-the-shelf” CAR-NK products. CD73-CAR engineering consistently enhanced cytotoxicity against PC-9 and H1299 cells with higher CD73 expression, but not CD73-low A549 cells, in peripheral blood-derived NK (PBNK), umbilical cord blood-derived NK (UBNK), and iPSC-derived NK (iNK) cells. Enhanced killing was consistently observed in both real-time cytotoxicity assays and three-dimensional spheroid models (Fig. 4a-n; Supplementary Fig. 4a-f). These findings indicate that CD73-CAR-mediated cytotoxicity is reproducible across multiple NK-cell sources and support further evaluation of the CD73-CAR strategy in clinically relevant NK-cell platforms.

### CD73-CAR-NK92 cells suppress tumor growth and prolong survival in CD73-high xenograft models

**Fig. 5:**
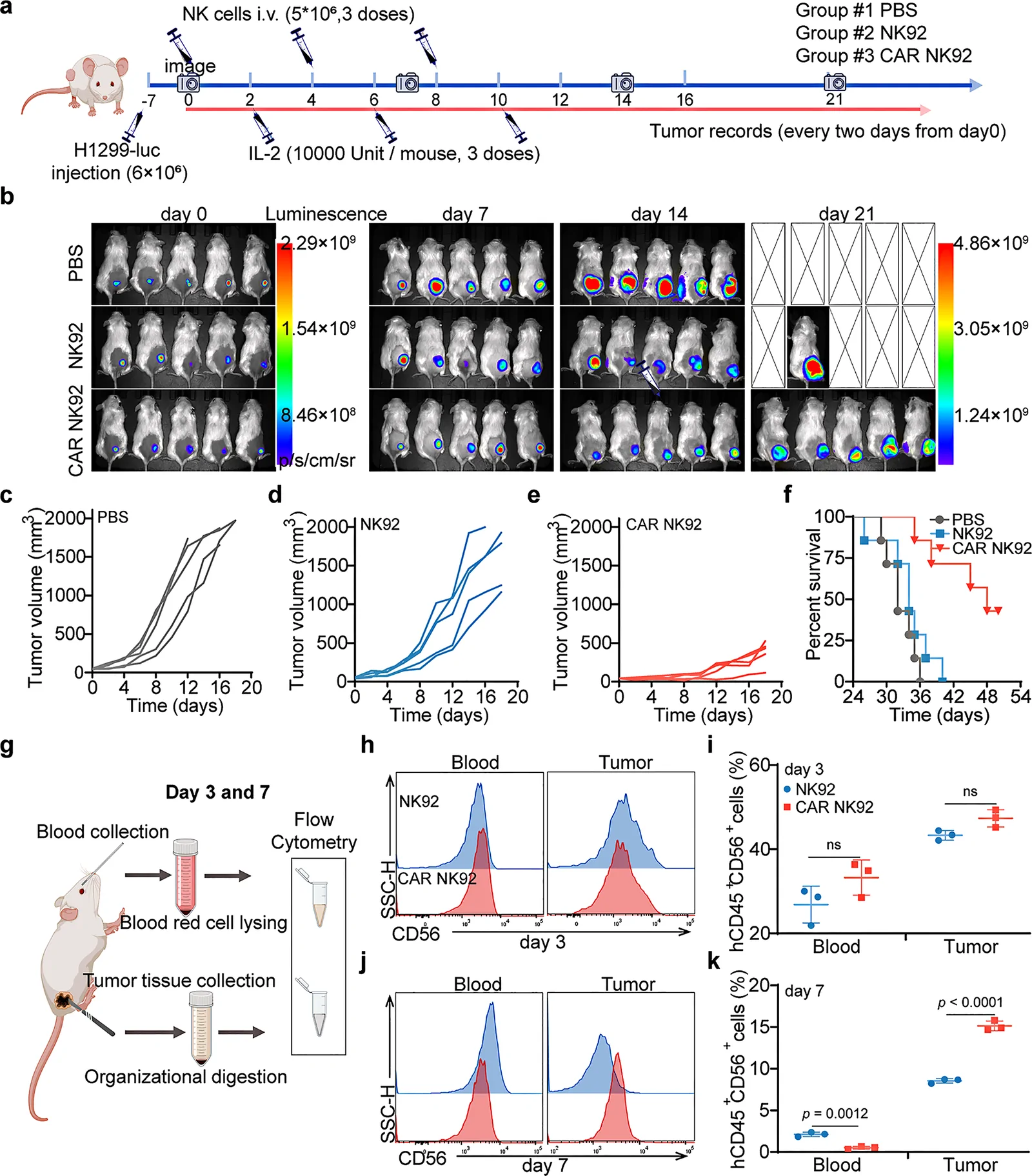
CD73-CAR‑NK92 cells suppress tumor growth and prolong survival in CD73⁺ xenograft models. **(a)** Schematic of the H1299‑luciferase subcutaneous CDX model and treatment regimen. **(b)** Longitudinal bioluminescence imaging of tumor burden in PBS, NK92, and CD73‑CAR‑NK92 treated mice on days 0, 7, 14, and 21. **(c-e)** Individual tumor growth curves of PBS **(c)**, NK92 **(d)**, and CD73‑CAR‑NK92 **(e)** treated mice. **(f)** Kaplan-Meier survival curves. **(g)** Schematic illustration of peripheral blood and tumor tissue collection. **(h**,** i)** Representative flow cytometry plots **(h)** and statistical quantification **(i)** of NK cell proportions in peripheral blood and tumor tissues at day 3 post‑treatment. **(j**,** k)** Representative flow cytometry plots **(j)** and statistical quantification **(k)** of NK cell proportions in peripheral blood and tumor tissues at day 7 post‑treatment. Data in **(i**,** k)** are presented as mean ± SD (*n* = 5-7 mice per group). For (**i**,** k**), a two-way ANOVA or mixed-effects model was used, followed by Šídák’s multiple comparisons test

Following the enhanced CD73-dependent activity observed in vitro, we next evaluated the in vivo antitumor efficacy of CD73-CAR-NK92 cells in an H1299 CDX model (Fig. 5a). Bioluminescence imaging showed rapid tumor progression in the PBS group, with all mice reaching ethical endpoints by day 21. NK92 treatment modestly delayed tumor growth, with only one mouse remaining alive at day 21. In contrast, CD73-CAR-NK92 treatment substantially suppressed tumor progression, with all mice remaining alive at day 21 (Fig. 5b). Consistent with these imaging findings, tumor volume measurements showed significantly slower tumor growth in the CD73-CAR-NK92 group than in both control groups (Fig. 5c-e), and Kaplan-Meier analysis confirmed prolonged survival following CD73-CAR-NK92 treatment (Fig. 5f).

We next examined the in vivo persistence and tumor accumulation of adoptively transferred NK cells. Peripheral blood and tumor tissues were collected on days 3 and 7 after treatment, respectively (Fig. 5g). Flow cytometric analysis showed a greater abundance of CD73-CAR-NK92 cells in peripheral blood at day 3 than parental NK92 cells (Fig. 5h, i). Similarly, greater accumulation of CD73-CAR-NK92 cells was observed in tumor tissues at day 7 (Fig. 5j, k). These findings are consistent with enhanced in vivo persistence and tumor-site accumulation of CD73-CAR-NK92 cells.

ELISA analysis at the experimental endpoint showed higher intratumoral levels of GZMB, TNF-α, and IFN-γ in the CD73-CAR-NK92 group than in the NK92 group (Supplementary Fig. 5a-c), consistent with enhanced local effector activity. Histological examination of the heart, liver, spleen, lung, and kidney revealed no overt pathological abnormalities during the observation period (Supplementary Fig. 5d).

Consistent results were obtained in a PC-9 xenograft model, in which CD73-CAR-NK92 treatment resulted in greater tumor suppression and prolonged survival compared with control treatments (Supplementary Fig. 6a-k). Together, these findings demonstrate reproducible antitumor activity of CD73-CAR-NK92 cells across two lung cancer CDX models, accompanied by enhanced in vivo persistence, tumor accumulation, and intratumoral effector activity.

### CD73-CAR-NK92 cells inhibit pulmonary metastatic progression

**Fig. 6:**
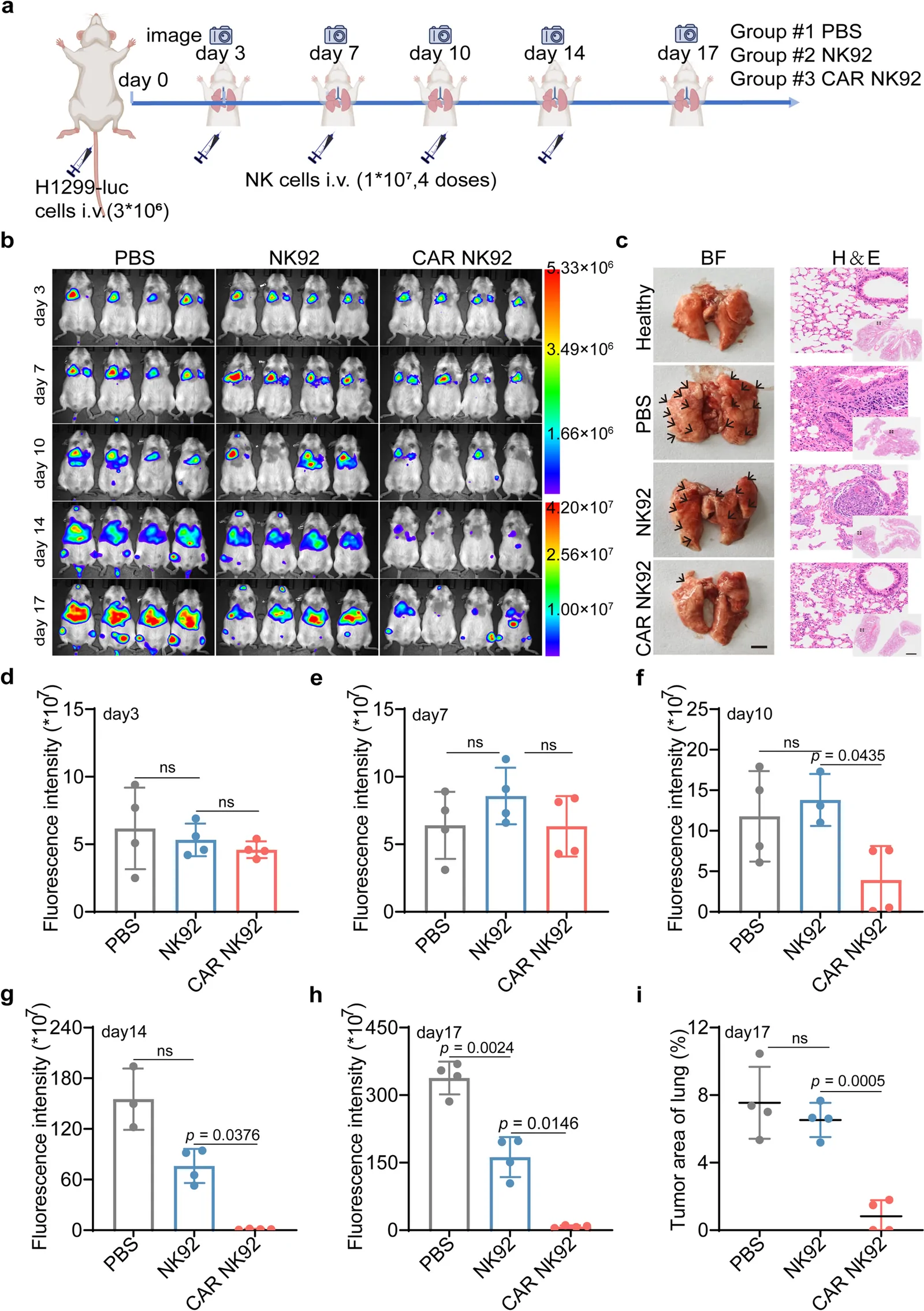
CD73-CAR-NK92 cells inhibit metastatic progression in a lung metastasis model. **(a)** Schematic of the H1299‑luc experimental lung metastasis model. **(b)** Serial bioluminescence images of pulmonary metastatic burden on days 3, 7, 10, 14, and 17 post‑tumor inoculation. **(c)** Representative gross images of lungs and corresponding H&E‑stained histological sections from healthy untreated mice and tumor‑bearing mice treated with PBS, NK92 cells, or CD73‑CAR‑NK92 cells at day 17. Arrows indicate metastatic nodules. Scale bar, 1 mm. **(d-h)** Quantification of bioluminescence signal intensity at days 3 **(d)**, 7 **(e)**, 10 **(f)**, 14 **(g)**, and 17 **(h)**. **(i)** Quantification of the tumor area fraction in lung sections based on H&E staining. Data in **(d-i)** are presented as mean ± SD (*n* = 4 mice per group). Statistical significance in (**d-i**) was assessed by one-way ANOVA with Welch’s correction (not assuming equal variances) followed by Dunnett’s T3 multiple comparisons test

Having established antitumor efficacy in subcutaneous models, we next evaluated the anti-metastatic activity of CD73-CAR-NK92 cells in an H1299-luciferase (H1299-luc) lung metastasis model (Fig. 6a), as metastatic dissemination remains the leading cause of mortality in LUAD patients [12]. Bioluminescence imaging performed three days after intravenous tumor-cell inoculation showed comparable pulmonary signals among the three groups, confirming successful model establishment and similar baseline tumor burden (Fig. 6b). Mice subsequently received PBS, NK92, or CD73-CAR-NK92 cells and were monitored twice weekly.

Longitudinal imaging showed progressive expansion of pulmonary tumor burden in both the PBS and NK92 groups over the observation period. In contrast, CD73-CAR-NK92 treatment resulted in a gradual and sustained reduction in luminescence signal intensity, with near-complete signal clearance in a subset of animals (Fig. 6b). Quantitative analysis at days 3, 7, 10, 14, and 17 further delineated the temporal dynamics of metastatic progression. At days 3 and 7, luminescence values were comparable among the three groups, indicating equivalent initial tumor engraftment. From day 10 onward, however, signals in the PBS and NK92 groups rose sharply, whereas those in the CD73-CAR-NK92 group steadily declined (Fig. 6d-h). Notably, The NK92 group showed a modestly slower increase than the PBS group, indicating limited antitumor activity of parental NK92 cells.

At day 17, lung tissues were collected for gross and histological evaluation. Numerous metastatic nodules were visible on the lung surfaces of PBS- and NK92-treated mice, whereas CD73-CAR-NK92-treated mice showed fewer and smaller lesions (Fig. 6c). Hematoxylin and eosin staining similarly showed extensive tumor involvement in the control groups and substantially reduced tumor burden following CD73-CAR-NK92 treatment. Quantification of tumor area confirmed a modest reduction in the NK92 group and a marked reduction in the CD73-CAR-NK92 group (Fig. 6i). Together, these findings demonstrate that CD73-CAR-NK92 treatment markedly inhibits pulmonary metastatic progression in this model.

### CD73-CAR-NK92 cells exhibit antitumor activity and local immune activation in R/R LUAD PDX models

**Fig. 7:**
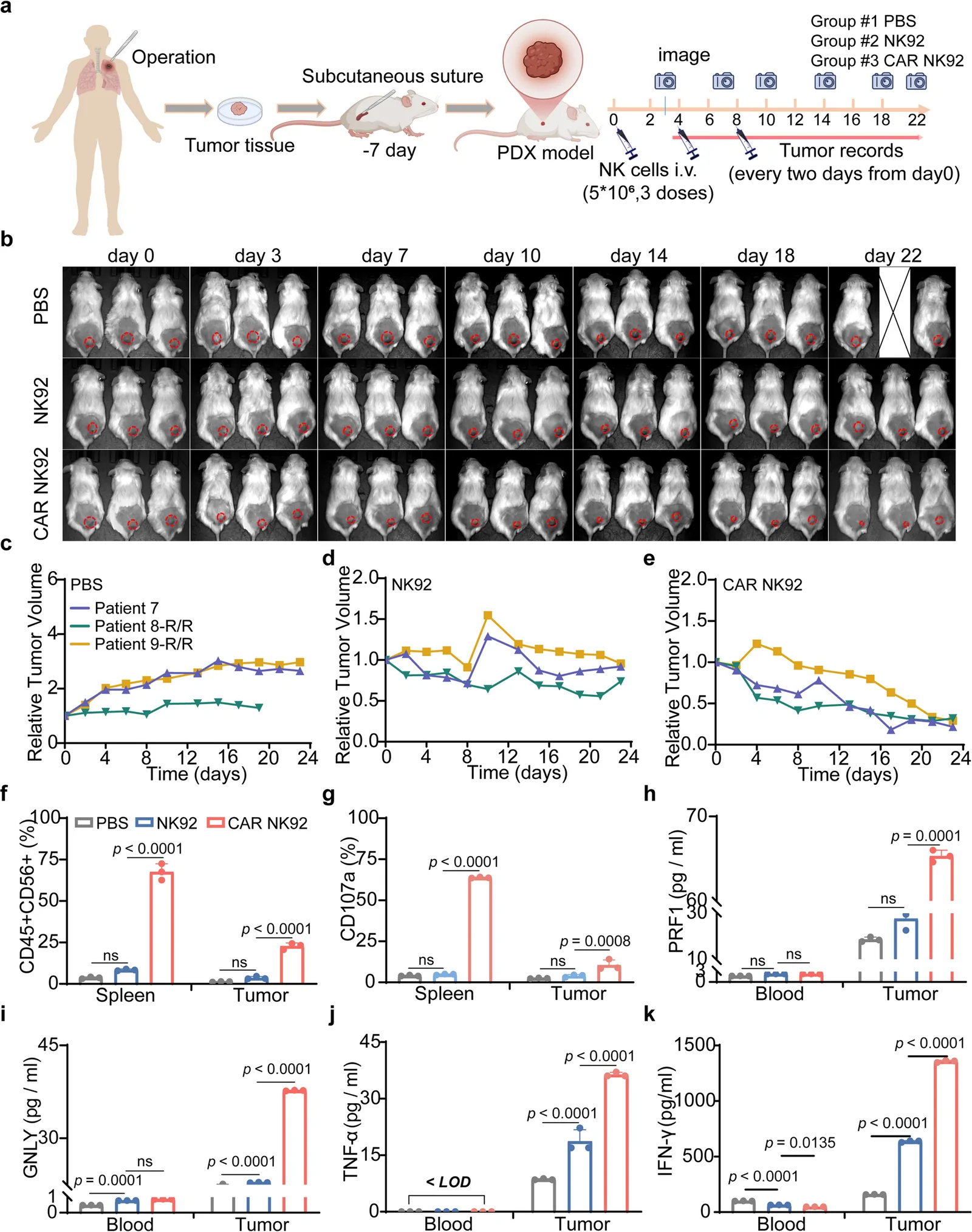
CD73-CAR-NK92 cells exhibit potent antitumor activity and local immune activation in an NCG-PDX model. **(a)** Schematic of PDX model establishment and treatment regimen. **(b)** Representative bright‑field images of tumor‑bearing mice over the treatment course. Tumor areas are outlined in red. **(c-e)** Individual tumor volume curves for PBS **(c)**, NK92 **(d)**, and CD73‑CAR‑NK92 **(e)** groups. **(f)** Proportions of NK cells in spleen and tumor tissues. **(g)** Percentages of CD107a⁺ activated NK cells in spleen and tumor. **(h-k)** ELISA quantification of PRF1 **(h)**, GNLY **(i)**, TNF‑α **(j)**, and IFN‑γ **(k)** in peripheral blood (serum) and tumor homogenates. < LOD, below limit of detection. Data in **(f-k)** are presented as mean ± SD (*n* = 3 mice per group). Statistical significance in (**f-k**) was determined using a two-way mixed-effects model followed by Šídák’s multiple comparisons test

We further evaluated the therapeutic activity of CD73-CAR-NK92 cells in a PDX model established by subcutaneous transplantation of tumor tissue from a patient with R/R LUAD (Fig. 7a). Tumors progressively enlarged in PBS-treated mice, whereas NK92 treatment modestly delayed tumor growth and CD73-CAR-NK92 treatment resulted in marked tumor regression (Fig. 7b). Longitudinal tumor-volume measurements showed a similar pattern, with continued growth in the PBS group, delayed progression with NK92 treatment, and sustained tumor reduction following CD73-CAR-NK92 treatment (Fig. 7c-e). At the experimental endpoint, tumors in the CD73-CAR-NK92 group were markedly smaller than those in the control groups (Supplementary Fig. 7a). Body weight remained stable across all groups during the observation period (Supplementary Fig. 7b).

We next examined NK-cell distribution following treatment. CD73-CAR-NK92-treated mice showed greater NK-cell abundance in both the spleen and tumor, with splenic NK-cell proportions approximately eightfold higher than those in the NK92 group and similarly increased accumulation within tumors (Fig. 7f). CD107a⁺ NK-cell frequencies were approximately tenfold higher in the spleen and 2.5-fold higher in tumors of CD73-CAR-NK92-treated mice than in NK92-treated mice (Fig. 7g). These findings indicate greater accumulation and activation of CD73-CAR-NK92 cells in both splenic and tumor compartments.

Consistent with these findings, intratumoral PRF1 and GNLY levels were significantly higher in the CD73-CAR-NK92 group than in the PBS and NK92 groups, while NK92 treatment produced modest increases relative to PBS (Fig. 7h, i). GZMB showed a similar pattern (Supplementary Fig. 7d). TNF-α was undetectable in peripheral blood, whereas intratumoral TNF-α levels were approximately twofold higher in the CD73-CAR-NK92 group than in the NK92 group (Fig. 7j). Peripheral IFN-γ levels remained low across all groups, while intratumoral IFN-γ was approximately threefold higher following CD73-CAR-NK92 treatment (Fig. 7k). Similarly, adenosine levels were reduced within tumors but remained unchanged in peripheral blood (Supplementary Fig. 7c). These findings indicate that the enhanced immune activity associated with CD73-CAR-NK92 treatment was predominantly localized to the tumor compartment, with low systemic cytokine levels during the observation period.

Collectively, these findings demonstrate marked antitumor activity of CD73-CAR-NK92 cells in an R/R LUAD PDX model, accompanied by increased NK-cell accumulation and activation, elevated intratumoral effector molecules, and reduced local adenosine levels.

### CD73-CAR-NK92 cells orchestrate robust local antitumor immunity in humanized immune system PDX models

**Fig. 8:**
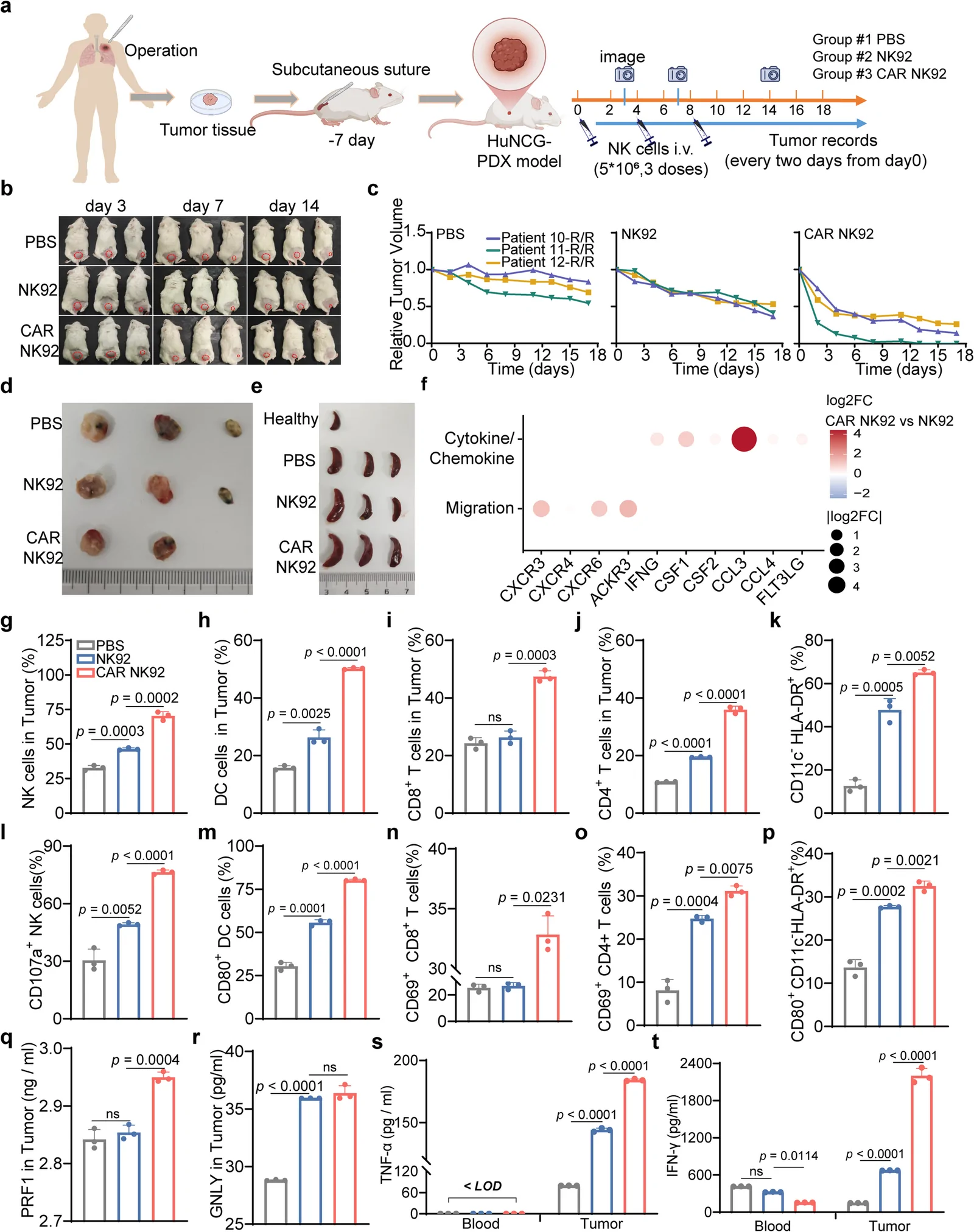
CD73-CAR NK92 cells orchestrate local antitumor immunity in an immune-humanized PDX model. **(a)** Schematic of model establishment and treatment regimen. **(b)** Representative bright‑field images of mice on days 3, 7, and 10 post‑treatment. Tumors are outlined in red. **(c)** Individual tumor growth curves for PBS, NK92, and CD73-CAR-NK92 groups. **(d-e)** Representative images of excised tumors **(d)** and spleens **(e)** at the experimental endpoint. **(f)** Bubble plot showing differentially expressed genes related to cytokine/chemokine signaling and migration between CD73-CAR-NK92 and parental NK92 cells, as identified by bulk RNA sequencing. Bubble size represents the absolute log2 fold change, and color intensity indicates the direction and magnitude of differential expression (log2FC, CAR-NK92 vs. NK92). **(g-j)** Flow cytometric quantification of tumor‑infiltrating NK cells **(g)**, dendritic cells **(h)**, CD8⁺ T cells **(i)**, and CD4⁺ T cells **(j)**. **(k)** Proportion of HLA^‑^DR⁺ monocyte/macrophages in spleens. **(l-o)** Percentages of activated cells among intratumoral NK cells **(l**, CD107a⁺**)**, dendritic cells **(m**, CD80⁺CD86⁺**)**, CD8⁺ T cells **(n**, CD69⁺**)**, and CD4⁺ T cells **(o**, CD69⁺**)**. **(p)** Proportion of CD80⁺ cells among splenic HLA‑DR⁺ monocyte/macrophages. **(q**,** r)** Intratumoral perforin **(q)** and granulysin **(r)** levels measured by ELISA. **(s**,** t)** TNF‑α **(s)** and IFN‑γ **(t)** concentrations in serum and tumor homogenates. < LOD, below limit of detection. Data in **(g-t)** are presented as mean ± SD (*n* = 3 mice per group). For **(g-r)**, statistical significance was assessed by one-way ANOVA followed by Tukey’s multiple comparisons test. For **(s**,** t)**, a two-way ANOVA or mixed-effects model was used, followed by Šídák’s multiple comparisons test

Finally, to evaluate the antitumor efficacy and safety of CD73-CAR-NK92 cell therapy in a human immune context, we established an immune-humanized PDX mouse model using R/R LUAD patient samples (Fig. 8a). Unlike conventional immunodeficient models, this system recapitulates key aspects of the human tumor-immune microenvironment, allowing clinically relevant evaluation of CD73-CAR-NK92 cell therapy. Representative bright-field images captured on days 3, 7, and 10 post-treatment revealed distinct patterns of tumor control. In the PBS group, modest tumor shrinkage was observed, likely attributable to the baseline antitumor activity of engrafted human immune cells. NK92 treatment produced more pronounced regression. CD73-CAR-NK92 cell administration induced rapid tumor dissipation, with marked tumor reduction observed as early as day 14 and complete clearance in several mice (Fig. 8b). Longitudinal tumor volume measurements corroborated these findings, demonstrating progressive growth attenuation across groups (Fig. 8c). At the experimental endpoint, CD73-CAR-NK92 cell-treated tumors were markedly smaller than controls, and a modest increase in spleen size was observed in the CAR cohort (Fig. 8d, e), suggesting active immune engagement.

Flow cytometric analysis of splenocytes revealed that human CD45⁺ engraftment was approximately 80% in both the NK92 and CD73-CAR-NK92 groups, compared with 16% in PBS-treated mice, and absolute CD45⁺ cell counts were highest in the CAR group (Supplementary Fig. 8a). Immunophenotyping demonstrated that CAR treatment led to a concurrent reduction of splenic NK cells, DCs, and CD4⁺ T cells (Supplementary Fig. 8b-d), which was accompanied by their enrichment within tumors. This inverse spleen–tumor relationship indicates active mobilization from lymphoid reservoirs, a pattern consistent with the bystander immune activation reported in clinical CAR-NK trials [40–42]. Consistent with a potential role in immune-cell recruitment, bulk RNA sequencing of CD73-CAR-NK92 cells identified increased expression of *CCL3*, *CSF1*, *CSF2*, *CXCR3*, *CXCR6*, and *ACKR3* (Fig. 8f). ELISA further showed increased secretion of CCL5 and CSF2 by CD73-CAR-NK92 and CD73-CAR-PBNK cells, particularly after H1299 co-culture, while CSF1 secretion was also increased in the PBNK system. CCL3 remained abundant across both NK92- and PBNK-based conditions (Supplementary Fig. 2i-o). In immune-humanized PDX mice, CCL3, CCL5, CSF1, and CSF2 were also elevated in tumor homogenates and serum following CD73-CAR treatment (Supplementary Fig. 8k-n). Conditioned medium from CD73-CAR-NK cultures promoted T-cell and dendritic-cell migration in Transwell assays, and this effect was significantly reduced by CCL3 neutralization (Supplementary Fig. 8o-r). These results support a role for soluble factors associated with CD73-CAR-NK treatment in immune-cell recruitment, with CCL3 directly contributing to the observed migratory response. The contributions of the other candidate mediators remain to be determined.

Flow-cytometric analysis further revealed increased infiltration of NK cells, DCs, CD8⁺ T cells, and CD4⁺ T cells in tumors following treatment, with activation-associated markers showing a similar pattern (Fig. 8g-j, l-o). These changes were accompanied by higher intratumoral PRF1 and GNLY levels (Fig. 8q, r). Within the myeloid compartment, CD11c⁺HLA-DR⁺ mature DCs were most abundant in tumors from the CAR-treated group, whereas CD11c⁻HLA-DR⁺ monocyte/macrophage populations were enriched in the spleen (Fig. 8h, k). Together with the increased CSF1 and CSF2 levels observed in vivo, these findings are consistent with altered myeloid-cell distribution and maturation following CAR-NK treatment, although the specific roles of CSF1 and CSF2 remain to be established.

A notable feature of CD73-CAR-NK92 therapy was the compartmentalization of inflammatory responses. Intratumoral TNF-α and IFN-γ were increased approximately twofold and fivefold, respectively, relative to NK92 controls (Fig. 8s, t), whereas peripheral cytokine levels remained low. During the 20-day observation period, serum IL-6, CRP, and hepatorenal function markers showed no treatment-related deterioration, and histological examination of major organs revealed no overt treatment-related abnormalities (Supplementary Fig. 9a-h). These results are consistent with the generally favorable early safety profile reported in clinical CAR NK studies [45, 46, 47–49].

In summary, using an immune-humanized PDX model derived from R/R LUAD patients, we demonstrated that CD73-CAR-NK92 therapy achieves robust tumor eradication, induces local immune cell infiltration and activation, and is associated with a compartmentalized safety profile. These integrated efficacy and safety data provide preclinical support for the translational potential of CD73-CAR-NK cell therapy in R/R LUAD.

## Discussion

R/R LUAD remains a difficult clinical problem with limited later-line options, and the modest objective response rates of current therapies emphasize the need for alternative strategies[1–4, 43–49]. CD73 is of particular interest because its expression rises progressively with LUAD malignancy and is highest in relapsed/refractory tumors, suggesting that it may be selected under therapeutic pressure [35]. Our study therefore examined whether CD73-directed CAR-NK cells could represent a clinically relevant approach for this population.

The antitumor activity we observed can be attributed to direct killing of CD73-high tumor cells together with the maintenance of effector function under adenosine-rich conditions. Functional evidence, including PKA activation and A2AR antagonist rescue experiments, indicates that the latter reflects resistance to adenosine-mediated inhibitory signaling at the level of NK cell function, rather than simply a secondary reduction in adenosine levels following tumor cell elimination. In addition, CD73-CAR-NK cells upregulated several chemokines and myeloid growth factors, and conditioned medium from these cells promoted T-cell and dendritic cell migration in vitro. These observations are consistent with a contribution of CAR-NK-derived factors to local immune cell recruitment and microenvironment remodeling, although the causal roles of individual mediators remain to be established.

Compared with CD73 antibodies or small-molecule inhibitors, which primarily block enzymatic activity, CD73-CAR-NK cells have the potential to eliminate CD73-positive tumor cells directly while also reducing the adenosine source. Although CD73 is broadly expressed in normal tissues, its lower expression in normal bronchial epithelium relative to tumor cells[19, 44–46], together with CAR-NK intrinsic safety advantages, enables preferential tumor targeting, as confirmed by the limited cytotoxicity of CD73-CAR-NK92 cells against normal BEAS-2B cells. Compared with CAR-T cells, the NK-based strategy showed a more compartmentalized pattern of immune activation, characterized by elevated intratumoral cytokines but low systemic levels, broadly consistent with the low rates of severe CRS, ICANS, and GvHD reported in clinical CAR-NK studies.

Several limitations should be acknowledged. First, the in vivo studies relied predominantly on non-irradiated NK92-derived cells. Because clinical NK92 products require irradiation before infusion, their persistence and therapeutic durability may differ from those observed in our models. In addition, systemic IL-2 support was used without an IL-2-only control, which limits interpretation of comparisons with the PBS group. The requirement for exogenous IL-2 may also limit the clinical translatability of the NK92-based model because systemic IL-2 administration is associated with clinically relevant toxicity. Although key findings were reproduced in vitro using PBNK, UBNK, and iNK cells, further in vivo validation using irradiated NK92 cells and alternative NK-cell sources is warranted. Second, the clinical cohort was relatively small, and the observed enrichment of CD73 in R/R LUAD requires confirmation in larger independent cohorts. Third, the safety data remain preliminary because of the limited animal numbers, short follow-up, and absence of comprehensive toxicological evaluation. In particular, potential on-target/off-tumor toxicity associated with CD73 expression in normal tissues requires further assessment. Fourth, the relatively small PDX and immune-humanized PDX cohorts limited the statistical power of these analyses. Although a patient-matched design was used to reduce inter-patient tumor heterogeneity, conventional randomization, tumor-burden stratification, and investigator blinding were not implemented in these cohorts. Human immune reconstitution was also supported by supplier-derived batch-level quality-control data rather than longitudinal monitoring of individual animals. Finally, whether CD73 antigen loss or downregulation may occur under long term CAR NK selective pressure is an important question warranting future investigation, as antigen escape has been recognized as one of the major resistance mechanisms limiting CAR T cell therapy in solid tumors [47–49].

In light of these limitations, future studies should evaluate CD73-CAR-NK cells derived from PBNK or iNK cells in clinically relevant in vivo models, as both sources are more clinically translatable than NK92 cells. Beyond monotherapy assessment, combining CD73-CAR-NK cells with clinically used monoclonal antibodies, such as PD-1/PD-L1 inhibitors or other standard therapeutic antibodies, should also be explored, as such combinations may enhance both direct tumor control and local immune activation. In addition, the individual contributions of candidate chemokines and growth factors to immune cell recruitment should be dissected using neutralizing antibodies or genetic approaches, and the potential emergence of CD73 antigen loss under sustained selective pressure should be monitored. Together, these directions would help define the optimal clinical positioning of CD73-CAR-NK therapy and facilitate its progression toward early-phase clinical evaluation in patients with CD73-positive R/R LUAD.

Taken together, these findings identify CD73 as a clinically relevant target in R/R LUAD and provide preclinical support for a CD73-directed CAR-NK strategy that combines direct tumor lysis, resistance to adenosine-mediated suppression, and preliminary evidence of local immune remodeling.

## Supplementary Information

Below is the link to the electronic supplementary material.

Supplementary Material 1

Supplementary Material 2

Supplementary Material 3

Supplementary Material 4

## Data Availability

The raw RNA-seq data are currently being deposited in a public repository, and the corresponding accession number will be added to the manuscript once it becomes available.
