# Supplementary material for: CD73-targeted CAR-NK cell therapy displays potent antitumor activity and translational potential in relapsed/refractory lung adenocarcinoma
Source: Biomark Res. 2026 Oct 28;14:154. doi: 10.1186/s40364-026-01036-5 (PMC13718019; doi:10.1186/s40364-026-01036-5)
Supplement: Supplementary file 1 [file 40364_2026_1036_MOESM1_ESM.docx]

**Supplementary Materials for**

**CD73-Targeted CAR-NK Cell Therapy displays Potent Antitumor Activity and Translational Potential in Relapsed/Refractory Lung Adenocarcinoma**

Qianqian Liu^1, 2#^, Qifei Dong^2, 3#^, Yimei Chen^4, 5, 6#^, Nannan Wang^2^, Dian Zhuang^1, 2^, Lihua Chen^7, 8^, Yanzhuo Ye^1, 2^, Shasha Huang^2^, Wenzhao Zhong^4*^, Wei Kevin Zhang ^2*^, Tao Xu^1, 2*^, Jiaqi Sun^2*^, Huisheng Liu^1, 2, 3*^

Correspondence to: [liu_huisheng@gzlab.ac.cn](mailto:liu_huisheng@gzlab.ac.cn); [sun_jiaqi@gzlab.ac.cn](mailto:sun_jiaqi@gzlab.ac.cn); [xu_tao@gzlab.ac.cn](mailto:xu_tao@gzlab.ac.cn); [zhang_wei@gzlab.ac.cn](mailto:zhang_wei@gzlab.ac.cn); [syzhongwenzhao@scut.edu.cn](mailto:syzhongwenzhao@scut.edu.cn)

**This PDF file includes:**

Materials and Methods

Supplementary Figures 1 to 9

Supplementary Tables 1-4

**Materials and Methods**

qPCR analysis

Total RNA was extracted using the RNeasy Plus Mini Kit (Qiagen, Cat#74106). Subsequently, 1 μg RNA was reversed transcription using the Maxima H Minus (Thermo, Cat#EP0753). The resulting cDNA was used as a template in quantitative polymerase chain reaction (qPCR) assays. The TB Green premix EX Taq Mix (TAKARA, Cat#RR820B) was used in an CFX96 Real-Time Detection instrument (BIO-RAD, USA) following the manufacturer protocol. Primers utilized in the qPCR were synthesized by Sangon Biotech Co., Ltd. (Guangzhou, China). Each sample was assayed in triplicate wells for qPCR analysis and the nuclear GAPDH gene served as the internal control. Data analysis was conducted using Graphpad Prism Software 8 and normalization was performed against undifferentiated H1299/A549 cells using ΔΔCt or relative to GAPDH using ΔCt.

Bulk RNA-seq processing and analysis

NK92 and CD73-CAR-NK92 cells were lysed using TRIzol (Thermo Fisher, Cat# 15596018) and flash frozen in liquid nitrogen for bulk RNA-seq analysis (two typical samples were taken from each group for mapping analysis). Quality testing, database construction and RNA sequencing were subsequently performed by Annoroad Gene Technology (Beijing) Co., Ltd. Raw sequencing reads (FASTQ ﬁles) are pre-processed, followed by alignment to the reference genome human genome GRCh38 using the alignment tool hisat2 (version 2.2.1). The sam ﬁles are further inspected, ﬁltered, and processed using samtools (version 1.13). HTseq counts obtained from bam ﬁles are annotated and subjected to DESeq2 (version 1.34.0) analysis in R (version 4.5.2). The genes marked in the heatmaps are indicated in the volcano plots according to their significant up- (red) or downregulation (blue). The Volcano plot reveled the top 9 pathways significantly enriched in the KEGG pathways.

**Enzyme-Linked Immunosorbent Assay (ELISA)**

For in vitro experiments, CD73-CAR-NK92 or NK92 cells were co-cultured with CD73-high tumor cells at effector-to-target (E: T) ratios of 0.5:1, 2:1, 4:1, and 8:1 for 5 hours. For primary clinical tumor samples, digested single-cell suspensions were co-cultured with effector cells under the same conditions. Cell-free culture supernatants were collected for detection. For in vivo samples, blood was collected via cardiac puncture, and serum was isolated by standing at room temperature for 2 h followed by centrifugation at 3000 g for 20 min. Tumor tissues were weighed and homogenized in ice-cold PBS supplemented with 1% protease inhibitor cocktail, then centrifuged at 15000 g for 20 min to collect the supernatant. The concentrations of adenosine, GZMB, IFN-γ, and TNF-α in all samples were determined using commercial ELISA kits (R&D Systems) according to the manufacturer’s instructions. For perforin detection, perforin-positive cells were quantified by flow cytometry after co-culture.All experiments were performed in three independent biological replicates.

**In vivo antitumor efﬁcacy and safety**

For the subcutaneous xenograft model, when tumor volume reached 100 mm³, mice were randomized into PBS, NK92, and CD73-CAR-NK92 groups. For the H1299-td-luc model, mice received 5×10⁶ NK92/CD73-CAR-NK92 cells or PBS. Human IL-2 (10,000 U) was injected after 2 days. Tumor size was measured every 2 days, with volume calculated as length × width² × 0.5.

For the lung metastasis xenograft model, treatment and imaging started on day 3 after tumor cell injection. Four doses of NK cells (1×10⁷) were given on days 3, 7, 10, and 14. Final imaging was performed on day 17, followed by euthanasia and lung dissection for comparison.

For the PDX model, patient tumor tissue was implanted on day -7. From day 0, tumor size was recorded every 2 days, with three NK cell treatments (1×10⁷) on days 0, 5, and 10. Monitoring continued until day 22.

For safety evaluation, peripheral blood was analyzed for blood routine and organ function. Major organs were harvested, fixed, sectioned, and stained with hematoxylin and eosin(H&E).

**Supplementary Figure 1.**

CAR construction and transcriptomic profiling.

**(a)** Plasmid sequencing chromatogram confirming the correct construction of the CD73-CAR lentiviral vector.

**(b)** Representative bright-field and corresponding green fluorescence images of NK92 cells following transduction with the CD73-CAR lentivirus, demonstrating successful viral infection and CAR expression.

**(c)** Flow cytometric analysis of fluorescent reporter expression in transduced NK92 cells, indicating CAR transduction efficiency.

**S****upplementary Figure 2.**

**(a)** KEGG pathway enrichment analysis of differentially expressed genes in CD73-CAR-NK92 versus NK92 cells. Bubble size indicates the number of DEGs in each pathway, and color represents the adjusted p-value (–log10).

**(b)** Summary of immune functional modules (Cytotoxicity, Activation, Migration, Cytokine/Chemokine, Inhibitory) for selected genes in NK92 and CD73-CAR-NK92 cells. Bubble size corresponds to the absolute log2 fold change, and color indicates the direction and magnitude of change (red, upregulated; blue, downregulated).

**(c–h)** Statistical quantification of the flow cytometry data presented in Figure 2g–l.

Bar graphs show mean fluorescence intensity (MFI) or percentage of positive cells for CD107a **(c)**, FASL **(d)**, NKG2D **(e)**, NKp46 **(f)**, TIGIT **(g)**, and NKG2A **(h)** on NK92 and CD73-CAR-NK92 cells following tumor co-culture.

Data are presented as mean ± SD (n = 3 independent experiments). Statistical significance was assessed by unpaired two-tailed Student's t-test.

**Supplementary Figure 3.**

Normal cell safety, adenosine resistance, 3D spheroid eradication, and antigen dependency validation.

**(a)** Cytotoxicity of NK92 and CD73-CAR-NK92 cells against normal bronchial epithelial BEAS-2B cells after 24 h co-culture at the indicated effector-to-target (E:T) ratios.

**(b, c)** Representative time-course images of PC-9 tumor spheroids **(b)** and PC9/A549 mixed spheroids **(c)** treated with PBS, NK92 cells, or CD73-CAR-NK92 cells. Scale bar, 200 μm.

**(d)** Extracellular adenosine levels measured by ELISA in supernatants after 6 h co‑culture of NK92 or CD73‑CAR‑NK92 cells with LUAD cell lines.

**(e, f)** Western blot analysis (e) and corresponding densitometric quantification using ImageJ (f) of CD73 protein expression in H1299 cells following siRNA-mediated knockdown.

**(g, h)** Western blot analysis **(g)** and corresponding densitometric quantification using ImageJ **(h)** of CD73 protein expression in A549 cells following lentiviral overexpression.

Data in (a, d, f, h) are presented as mean ± SD (n = 3 independent experiments). Statistical significance was assessed by unpaired two-tailed Student's t-test.

**Supplementary Figure 4.**

3D spheroid assays with diverse NK sources.

**(a, b)** Representative bright-field images of H1299 **(a)** and PC9 **(b)** tumor spheroids treated with iNK cells or CAR-iNK cells.

**(c)** Representative images of H1299/A549 mixed spheroids treated with UBNK cells or CAR-UBNK cells, showing preferential elimination of CD73 ⁺ ⁺ H1299 cells.

**(d–f)** Representative images of A549 spheroids **(d)**, H1299 spheroids **(e)**, and H1299/A549 mixed spheroids **(f)** treated with PBNK cells or CAR-PBNK cells.

All scale bars, 200 μm. Images are representative of three independent experiments.

**Supplementary Figure 5.**

Intratumoral effector molecule levels and systemic toxicity assessment in the H1299 CDX model.

**(a–c)** ELISA quantification of intratumoral granzyme B **(a)**, TNF-α **(b)**, and IFN-γ **(c)** levels in tumor tissues harvested at the experimental endpoint from PBS-, NK92-, and CD73-CAR NK92-treated mice.

**(d)** Representative H&E staining images of heart, liver, spleen, lung, and kidney sections from NSG mice in the indicated treatment groups. Scale bar, 20 μm.

Data in **(a–c)** are presented as mean ± SD (n = 5-7 mice per group). Statistical significance was assessed by one-way ANOVA with Tukey's post hoc test.

**Supplementary Figure 6.**

Antitumor efficacy in the PC9 CDX model.

**(a)** Treatment schema of the PC9 subcutaneous CDX model.

**(b)** Longitudinal bioluminescence imaging of tumor burden in PBS, NK92, and CD73-CAR NK92 treated mice on days 0, 7, 14, 23, and 35 post-tumor inoculation.

**(c–e)** Individual tumor growth curves of mice in the PBS **(c)**, NK92 **(d)**, and CD73-CAR NK92 **(e)** groups.

**(f)** Kaplan–Meier survival curves of treated mice.

**(g)** Schematic illustration of peripheral blood and tumor tissue collection.

**(h, i)** Flow cytometric quantification of NK cell proportions in peripheral blood **(h)** and tumor tissues **(i)** at day 3.

**(j, k)** Flow cytometric quantification of NK cell proportions in peripheral blood **(j)** and tumor tissues **(k)** at day 7.

Data in **(c–f)** are presented as mean ± SD (n = 5-7 mice per group). Statistical significance in **(f)** was determined by the log-rank test; in **(c-e)** by unpaired two-tailed Student's t-test.

**Supplementary Figure 7.**

Endpoint analyses of NCG-PDX model.

**(a)** Representative images of excised tumors from NCG-PDX mice at the experimental endpoint in PBS, NK92, and CD73-CAR-NK92 treatment groups.

**(b)** Body weight curves of mice monitored throughout the treatment period.

**(c)** Intratumoral adenosine concentrations measured by ELISA in tumor homogenates from the indicated groups.

**(d)** Intratumoral GZMB levels measured by ELISA.

Data in **(b–d)** are presented as mean ± SD (n = 3–5 mice per group). Statistical significance was assessed by unpaired two-tailed Student's t-test.

**Supplementary Figure 8.**

Immune profiling of splenic populations and intratumoral effector molecules in the humanized PDX model.

**(a)** Representative flow cytometry gating strategy for splenic immune cells. Top row: FSC-SSC gate identifying the major splenic cell population. Middle row: DAPI⁻ gate excluding dead cells. Bottom row: CD45⁺ gate identifying human immune cells.

**(b–d)** Flow cytometric quantification of splenic NK cells **(b)**, dendritic cells **(c)**, and CD8⁺ T cells **(d)**.

**(f–i)** Proportions of activated cells among splenic NK cells (**f**, CD107a⁺), dendritic cells (**g**, CD80⁺), CD8⁺ T cells (**h**, CD69⁺), and CD4⁺ T cells (**i**, CD69⁺).

**(j)** ELISA quantification of Adenosine concentrations in tumor homogenates.

**(k)** Representative H&E staining images of spleen sections from healthy mice and tumor-bearing mice treated as indicated. Scale bar, 20 μm.

Data in (**b–d, f–j**) are presented as mean ± SD (n = 3 mice per group). Statistical significance was assessed by unpaired two-tailed Student's t-test.

**Supplementary Figure 9.**

Systemic safety and organ function assessment in the humanized PDX model.

**(a–e)** Serum levels of AST **(a)**, ALT **(b)**, creatinine **(c)**, uric acid **(d)**, and Fe **(e)** in healthy mice (n = 1, serving as a non-tumor baseline reference) and tumor-bearing mice treated with PBS (n = 3), NK92 cells (n = 3), or CD73-CAR NK92 cells (n = 3).

**(f, g)** Serum concentrations of IL-6 **(f)** and C-reactive protein **(g)** measured by ELISA in the indicated groups.

**(h)** Representative H&E staining images of heart, liver, spleen, lung, and kidney sections from healthy mice and tumor-bearing mice treated as indicated. Scale bar, 20 μm.

Data from tumor-bearing groups in **(a–g)** are presented as mean ± SD. Statistical comparisons among PBS, NK92, and CD73-CAR NK92 groups in **(a–e)** were performed by one-way ANOVA with Tukey's post hoc test; in **(f, g)** by unpaired two-tailed Student's t-test. Due to the single-sample nature of the healthy group, no statistical comparisons were made against this baseline reference.

**Supplementary Table 1.**

Primers used for qPCR

| Gene | Forward | Reverse |
| --- | --- | --- |
| *CD73* | ATTCTAGAGCTAGCGAATTCATGTGTCCCCGAGCCGCG | CCGTCATGGTCTTTGTAGTCGGATCCCTATTGGTATAAAC |
| *h-GAPDH* | TGCACCACCAACTGCTTAGC | GGCATGGACTGTGGTCATGAG |

**Supplementary Table 2.**

Antibodie**s**

| Primary antibodies | |  |  |  |
| --- | --- | --- | --- | --- |
| Antigen | **Species** | **Cat.No.** | **Company** | **Dilution** |
| *CD73* | Rabbit | ab317462 | abcam | 1:500(IF)  1:2000(WB) |

| Second antibodies |  | | |
| --- | --- | --- | --- |
| Antigen | **Cat.No.** | **Company** | **Dilution** |
| HRP-conjugated Goat Anti-Rabbit IgG(H+L) | SA00001-2 | Proteintech | 1:5000(WB) |
| HRP-conjugated Goat Anti-Mouse IgG(H+L) | SA00001-1 | Proteintech | 1:5000(WB) |
| Donkey anti-Rabbit IgG Alexa Fluor 546 | A10040 | Invitroge™ | 1:500(IF) |

**Supplementary Table 3.**

FCM antibodies

| Antigen | Fluorochrome | Cat.No. | Company | Dilution |
| --- | --- | --- | --- | --- |
| *CD73* | FITC | 344015 | BioLegend | 1:100 |
| *CD56* | PE | 304606 | Biolegend | 1:100 |
| *CD107a* | APC/Cyanine7 | 328629 | Biolegend | 1:100 |
| *FasL* | APC | 306421 | Biolegend | 1:100 |
| *NKG2D* | PE/Cyanine7 | 320811 | Biolegend | 1:100 |
| *NKp46* | FITC | G3061 | Selleck | 1:100 |
| *TIGIT* | APC/Cyanine7 | 372734 | Biolegend | 1:100 |
| *NKG2A* | APC | 375108 | Biolegend | 1:100 |
| *CD45* | APC | H0093 | Selleck | 1:100 |
| *CD4* | PE/Cyanine7 | 344611 | BioLegend | 1:100 |
| *CD8* | FITC | 344703 | BioLegend | 1:100 |
| *CD69* | PE | 310906 | BioLegend | 1:100 |
| *CD80* | PE/Cyanine7 | L307.4 | Selleck | 1:100 |

**Supplementary Table 4**

List of Abbreviations

| Abbreviation | Full Name |
| --- | --- |
| ACKR3 | Atypical Chemokine Receptor 3 |
| ALT | Alanine Aminotransferase |
| AST | Aspartate Aminotransferase |
| CAR | Chimeric Antigen Receptor |
| CCL3 | C‑C Motif Chemokine Ligand 3 |
| CCL4 | C‑C Motif Chemokine Ligand 4 |
| CCL5 | C‑C Motif Chemokine Ligand 5 |
| CDX | Cell line‑Derived Xenograft |
| CRP | C‑Reactive Protein |
| CRS | Cytokine Release Syndrome |
| CSF1 | Colony Stimulating Factor 1 |
| CSF2 | Colony Stimulating Factor 2 |
| CXCR3 | C‑X‑C Motif Chemokine Receptor 3 |
| CXCR6 | C‑X‑C Motif Chemokine Receptor 6 |
| DC | Dendritic Cell |
| ELISA | Enzyme‑Linked Immunosorbent Assay |
| EMT | Epithelial‑to‑Mesenchymal Transition |
| E:T | Effector‑to‑Target ratio |
| FASLG | Fas Ligand |
| GNLY | Granulysin |
| GvHD | Graft‑versus‑Host Disease |
| GZMA | Granzyme A |
| GZMB | Granzyme B |
| HLA‑DR | Human Leukocyte Antigen‑DR |
| ICANS | Immune Effector Cell‑Associated Neurotoxicity Syndrome |
| IFN‑γ | Interferon‑Gamma |
| iNK | iPSC‑Derived Natural Killer cell |
| iPSC | Induced Pluripotent Stem Cell |
| KEGG | Kyoto Encyclopedia of Genes and Genomes |
| LUAD | Lung Adenocarcinoma |
| MDSC | Myeloid‑Derived Suppressor Cell |
| NCG | NOD‑Prkdc^scid Il2rg^tm1 mice |
| NK | Natural Killer cell |
| NSCLC | Non‑Small Cell Lung Cancer |
| PBNK | Peripheral Blood‑Derived Natural Killer cell |
| PDX | Patient‑Derived Xenograft |
| PFS | Progression‑Free Survival |
| PI3K‑AKT | Phosphoinositide 3‑Kinase‑AKT |
| PRF1 | Perforin‑1 |
| R/R | Relapsed/Refractory |
| scFv | Single‑Chain Variable Fragment |
| TIGIT | T‑cell Immunoreceptor with Ig and ITIM domains |
| TME | Tumor Microenvironment |
| TNF‑α | Tumor Necrosis Factor‑Alpha |
| Treg | Regulatory T cell |
| UBNK | Umbilical Cord Blood‑Derived Natural Killer cell |
