# Supplementary material for: CD73-targeted CAR-NK cell therapy displays potent antitumor activity and translational potential in relapsed/refractory lung adenocarcinoma
Source: Biomark Res. 2026 Oct 28;14:154. doi: 10.1186/s40364-026-01036-5 (PMC13718019; doi:10.1186/s40364-026-01036-5)
Supplement: Supplementary file 2 [file 40364_2026_1036_MOESM2_ESM.docx]

**Figure1.** CD73 is upregulated in relapsed/refractory LUAD and serves as a rational target for CAR NK therapy.

**(f)** Western blot analysis of CD73 protein expression in LUAD cell lines and BEAS-2B cells.

CD73

70 KDa

β-actin

42 KDa

**Supplementary Figure 3.**

Normal cell safety, adenosine resistance, 3D spheroid eradication, and antigen dependency validation.

**(e)**Western blot analysis of CD73 protein expression in H1299 cells following siRNA-mediated knockdown.

CD73

70 KDa

GAPDH

36 KDa

**Supplementary Figure 3.**

Normal cell safety, adenosine resistance, 3D spheroid eradication, and antigen dependency validation.

**(g)** Western blot analysis of CD73 protein expression in A549 cells following lentiviral overexpression.

GAPDH

36 KDa

CD73

70 KDa
