# Supplementary material for: CD73-targeted CAR-NK cell therapy displays potent antitumor activity and translational potential in relapsed/refractory lung adenocarcinoma
Source: Biomark Res. 2026 Oct 28;14:154. doi: 10.1186/s40364-026-01036-5 (PMC13718019; doi:10.1186/s40364-026-01036-5)
Supplement: Supplementary file 3 [file 40364_2026_1036_MOESM3_ESM.docx]

**Figure1.** CD73 is upregulated in relapsed/refractory LUAD and serves as a rational target for CAR NK therapy.

**(f)** Western blot analysis of CD73 protein expression in LUAD cell lines and BEAS-2B cells.

CD73

70 KDa

β-actin

42 KDa

**Supplementary Figure 3.**

Normal cell safety, adenosine resistance, 3D spheroid eradication, and antigen dependency validation.

**(e)**Western blot analysis of CD73 protein expression in H1299 cells following siRNA-mediated knockdown.

CD73

70 KDa

GAPDH

36 KDa

**Supplementary Figure 3.**

Normal cell safety, adenosine resistance, 3D spheroid eradication, and antigen dependency validation.

**(g)** Western blot analysis of CD73 protein expression in A549 cells following lentiviral overexpression.

GAPDH

36 KDa

CD73

70 KDa

**Figure 3.**

CD73-CAR-NK cells mediate antigen-dependent cytotoxicity and resist adenosine-mediated suppression.

**(m)** Western blot analysis of phosphorylated PKA substrate levels in NK92, and CD73 CAR NK92 cells under the indicated conditions (untreated, exogenous adenosine, or H1299 co culture).

Phosphorylated PKA substrate

GAPDH

36 KDa

**Supplementary Figure 3.**

Normal cell safety, adenosine resistance, 3D spheroid eradication, and antigen dependency validation.

**(i)** Western blot analysis of phosphorylated PKA substrate levels in PBNK and CD73-CAR-PBNK cells following stimulation with exogenous adenosine or co-culture with H1299 cells.

Phosphorylated PKA

substrate

GAPDH

36 KDa

**Supplementary Figure 3 (Left) and Figure 3 (Right).**

CD73-CAR-NK cells mediate antigen-dependent cytotoxicity and resist adenosine-mediated suppression.

**(i)** Western blot analysis of phosphorylated PKA substrate levels in PBNK and CD73-CAR-PBNK cells following stimulation with exogenous adenosine or co-culture with H1299 cells **(Left)**.

**(m)** Western blot analysis of phosphorylated PKA substrate levels in NK92, and CD73 CAR NK92 cells under the indicated conditions (untreated, exogenous adenosine, or H1299 co culture) **(Right)**.

Total PKA

42KDa
