# Supplementary material for: CD73-targeted CAR-NK cell therapy displays potent antitumor activity and translational potential in relapsed/refractory lung adenocarcinoma
Source: Biomark Res. 2026 Oct 28;14:154. doi: 10.1186/s40364-026-01036-5 (PMC13718019; doi:10.1186/s40364-026-01036-5)
Supplement: Supplementary file 4 [file 40364_2026_1036_MOESM4_ESM.docx]

**Supplementary Materials for**

**CD73-Targeted CAR-NK Cell Therapy Displays Potent Antitumor Activity and Translational Potential in Relapsed/Refractory Lung Adenocarcinoma**

Qianqian Liu^1, 2#^, Qifei Dong^2, 3#^, Yimei Chen^4, 5, 6#^, Nannan Wang^2^, Dian Zhuang^1, 2^, Lihua Chen^7, 8^, Yanzhuo Ye^1, 2^, Shasha Huang^2^, Wenzhao Zhong^4*^, Wei Kevin Zhang ^2*^, Tao Xu^1, 2*^, Jiaqi Sun^2*^, Huisheng Liu^1, 2, 3*^

Correspondence to: [liu_huisheng@gzlab.ac.cn](mailto:liu_huisheng@gzlab.ac.cn); [sun_jiaqi@gzlab.ac.cn](mailto:sun_jiaqi@gzlab.ac.cn); [xu_tao@gzlab.ac.cn](mailto:xu_tao@gzlab.ac.cn); [zhang_wei@gzlab.ac.cn](mailto:zhang_wei@gzlab.ac.cn); [syzhongwenzhao@scut.edu.cn](mailto:syzhongwenzhao@scut.edu.cn)

**This PDF file includes:**

Materials and Methods

Supplementary Figures 1-9

Supplementary Tables 1-6

**Materials and Methods**

**Cell lines**

Human non-small cell lung cancer cell lines (H1299, PC9, A549), human lung squamous cell carcinoma cell line SK-MES-1, human bronchial epithelial cell line BEAS-2B, HEK-293T cells, and human natural killer cell line NK92 derived from malignant non-Hodgkin's lymphoma were stored in Guangzhou National Laboratory. CD73-CAR-NK92, NK92-EGFP, A549-EGFP, H1299-td-luc, and PC-9-td-luc cell lines were generated via lentivirus transduction. All cell lines were authenticated by short tandem repeat (STR) profiling and tested negative for mycoplasma contamination before use. Lung cancer cell lines were cultured in Roswell Park Memorial Institute (RPMI) 1640 medium (Gibco) supplemented with 10% fetal bovine serum (FBS) and 1% penicillin-streptomycin under standard conditions (37°C, 5% CO₂). Other adherent cell lines were cultured in DMEM (Gibco) medium containing 10% fetal bovine serum and 1% penicillin-streptomycin. Peripheral blood-derived natural killer (PBNK) cells were purchased from Shenzhen Cell Valley Biotechnology Co., Ltd., China, and maintained in the manufacturer's proprietary NK cell culture medium. Umbilical cord blood-derived natural killer (UBNK) cells were purchased from Guangzhou Muran Biochemical Technology Co., Ltd., China, and maintained in the manufacturer's proprietary NK cell culture medium. Peripheral blood mononuclear cells (PBMCs) were obtained from Aoneng Biotechnology Co., Ltd., China, and maintained in RPMI 1640 medium supplemented with 10% FBS during the experimental period. NK92 and CD73-CAR-NK92 cells were cultured in NK-92 Cell Complete Medium (Procell Life Science & Technology) supplemented with 100 U/mL recombinant human interleukin-2 (IL-2, Novoprotein). All cells were statically cultured in a constant temperature and humidity incubator at 37 °C with 5% CO₂, and the medium was changed and the cells were passaged regularly to maintain the logarithmic growth phase of the cells.

**Immunofluorescence staining**

Immunofluorescence (IF) staining was performed on adherent cells including H1299, A549, PC-9, SK, and BEAS-2B cells. Cells were fixed with 4% paraformaldehyde at room temperature for 15–30 minutes, followed by permeabilization and blocking with QuickBlock immunostaining blocking solution for 50 minutes. Subsequently, the cells were incubated with primary anti-CD73 antibody (1:1000, Abcam) overnight at 4°C, and then with Alexa Fluor 594-conjugated secondary antibody (1:500, Invitrogen™) for 1 hour at room temperature. Nuclei were counterstained with DAPI. Images were captured using a confocal laser scanning microscope, and the mean fluorescence intensity of CD73 was quantified using ImageJ software.

**qPCR analysis**

Total RNA was extracted using the RNeasy Plus Mini Kit (Qiagen, Cat#74106). Subsequently, 1 μg RNA was reversed transcription using the Maxima H Minus (Thermo, Cat#EP0753). The resulting cDNA was used as a template in quantitative polymerase chain reaction (qPCR) assays. The TB Green premix EX Taq Mix (TAKARA, Cat#RR820B) was used in an CFX96 Real-Time Detection instrument (BIO-RAD, USA) following the manufacturer protocol. Primers utilized in the qPCR were synthesized by Sangon Biotech Co., Ltd. (Guangzhou, China). Each sample was assayed in triplicate wells for qPCR analysis and the nuclear GAPDH gene served as the internal control. Data analysis was conducted using Graphpad Prism Software 8 and normalization was performed against undifferentiated H1299/A549 cells using ΔΔCt or relative to GAPDH using ΔCt.

**Validation of CD73 knockdown and overexpression**

For siRNA-mediated CD73 knockdown, CD73 mRNA and protein expression were assessed by qPCR and western blotting, respectively. qPCR was performed 24 h after siRNA transfection, and western blotting was performed at 48 h. Functional assays were initiated 24 h after transfection and completed within the following 24 h. For CD73 overexpression, cells were transduced with lentivirus, and CD73 protein expression was assessed by western blotting before functional assays. Expression was reassessed after the assays to verify maintenance during the experimental period.

**Public transcriptomic and survival analyses**

LUAD survival data stratified by CD73 expression were obtained from The Human Protein Atlas database, comprising 78 patients in the CD73-low group and 28 patients in the CD73-high group. For transcriptomic comparison, FPKM-normalized RNA-sequencing data were obtained from the GDC TCGA Lung Adenocarcinoma (LUAD) cohort through the UCSC Xena platform. The analysis included normal lung tissues (n = 58), primary LUAD tumors (n = 528), and recurrent LUAD tumors (n = 2). Because of the limited number of recurrent samples, this analysis was considered exploratory and descriptive.

**Bulk RNA-seq processing and analysis**

NK92 and CD73-CAR-NK92 cells were collected for bulk RNA-seq analysis, with four independent biological replicates per group. mRNA was enriched using oligo(dT) magnetic beads, followed by library preparation and quality control. Libraries were sequenced on a DNBSEQ-T7 platform using paired-end 150-bp reads. An average of approximately 45.9 million clean reads per sample was obtained (range, 41.7–50.6 million reads), with Q30 values >90%. Clean reads were aligned to the human GRCh38 reference genome using HISAT2 (v2.2.1), with overall mapping rates of 95.25–96.30%. Gene-level read counts were generated using HTSeq (v2.0.2), and differential expression analysis was performed using DESeq2 (v1.18.1) with Benjamini–Hochberg FDR correction. DEGs were defined as |log₂ fold change| ≥ 1 and adjusted P < 0.05.

**Enzyme-Linked Immunosorbent Assay (ELISA)**

For in vitro assays, CD73-CAR-NK92 cells or control NK92 cells were co-cultured with CD73-high tumor cells at effector-to-target (E:T) ratios of 0.5:1, 2:1, 4:1, and 8:1. After 6 h of co-culture, cell-free culture supernatants were collected from the indicated E:T ratios for the measurement of PRF1, GZMB, IFN-γ, and TNF-α. For CCL3, CCL5, CSF1, and CSF2, culture supernatants were collected after 6 h of co-culture at an E:T ratio of 4:1. For adenosine quantification, co-culture was continued until the end of the cytotoxicity assay (24 h), and cell-free culture supernatants were then collected. Supernatants were analyzed immediately or stored at −80°C until analysis.

For in vivo samples, blood was collected via cardiac puncture, and serum was isolated by standing at room temperature for 2 h followed by centrifugation at 3000 × g for 20 min. Tumor tissues were weighed and homogenized in ice-cold PBS supplemented with 1% protease inhibitor cocktail, then centrifuged at 15,000 × g for 20 min to collect the supernatant.

The concentrations of adenosine, PRF1, GZMB, IFN-γ, TNF-α, CCL3, CCL5, CSF1, and CSF2 in culture supernatants, serum, and tumor homogenates were determined using commercial ELISA kits (R&D Systems) according to the manufacturer's instructions. All experiments were performed in three independent biological replicates.

**Transwell migration assay**

To evaluate the chemotactic capacity of CAR-NK-derived soluble factors, NK92, CD73-CAR-NK92, PBNK, and CD73-CAR-PBNK cells were cultured alone or co-cultured with H1299 cells at an effector-to-target ratio of 4:1 for 24 h. Cell-free conditioned medium was collected, centrifuged, and filtered, then diluted 1:1 with chemotaxis buffer (RPMI 1640 containing 0.5% BSA). Peripheral blood mononuclear cells (PBMCs) were resuspended in chemotaxis buffer at 3.5 × 10⁶ cells/mL, and 100 μL of the cell suspension (3.5 × 10⁵ cells) was added to the upper chamber of a Transwell insert with a 5-μm pore size (Corning). The lower chamber contained 600 μL of diluted conditioned medium. For neutralization experiments, an anti-human CCL3 neutralizing antibody (1 μg/mL) was added to the conditioned medium of the CD73-CAR co-culture group and pre-incubated for 30 min before addition to the lower chamber. After incubation for 6 h at 37°C in 5% CO₂, cells that migrated into the lower chamber were collected for flow cytometric analysis. The proportions of migrated T cells and dendritic cells were determined by staining with anti-CD3 and anti-CD11c antibodies. Experiments were performed in triplicate and repeated three times independently.

**In vivo antitumor efﬁcacy and safety**

For the subcutaneous xenograft model, NSG mice bearing H1299‑td‑luc or PC‑9‑td‑luc tumors were used. When tumor volume reached approximately 100 mm³, mice were assigned to PBS, NK92, or CD73‑CAR‑NK92 groups. Mice received 5 × 10⁶ NK92 cells, 5 × 10⁶ CD73‑CAR‑NK92 cells, or an equivalent volume of PBS. To support NK cell survival, all groups received intraperitoneal injection of human IL‑2 (10,000 U per mouse) every other day, starting two days after the first effector cell administration. Tumor size was measured every 2 days, and tumor volume was calculated as length × width² × 0.5.

For the lung metastasis model, female NSG mice were injected intravenously with 3 × 10⁶ H1299‑td‑luc cells on day 0. Starting on day 3, mice received four intravenous doses of NK92 cells or CD73‑CAR‑NK92 cells (1 × 10^7^ per dose) on days 3, 7, 10, and 14, or PBS as control. IL‑2 support was given as described above. Bioluminescence imaging was performed on days 3, 7, 10, 14, and 17, and lungs were harvested on day 17 for gross and histological evaluation.

For the PDX model, fresh tumor tissue from each patient was divided into three portions and subcutaneously implanted into three mice, which were assigned to the PBS, NK92, and CD73‑CAR‑NK92 groups, respectively. This patient‑matched design was used to minimize baseline tumor burden differences among groups. Then, the mice received three doses of NK92 cells or CD73‑CAR‑NK92 cells (5 × 10^6^ per dose) on days 0, 5, and 10, or PBS as control. Tumor size was recorded every 2 days until day 22.

For safety evaluation, peripheral blood samples were collected for routine hematology and organ function analyses. Major organs, including heart, liver, spleen, lung, and kidney, were harvested, fixed, sectioned, and stained with hematoxylin and eosin (H&E).

**Supplementary Figure 1.**

CAR construction and transcriptomic profiling.

**(a)** Plasmid sequencing chromatogram confirming the correct construction of the CD73-CAR lentiviral vector.

**(b)** Representative bright-field and corresponding green fluorescence images of NK92 cells following transduction with the CD73-CAR lentivirus, demonstrating successful viral infection and CAR expression.

**(c)** Flow cytometric analysis of fluorescent reporter expression in transduced NK92 cells, indicating CAR transduction efficiency.

**S****upplementary Figure 2.**

Transcriptomic and functional characterization of CD73-CAR-NK cells.

**(a)** KEGG pathway enrichment analysis of differentially expressed genes in CD73-CAR-NK92 versus NK92 cells. Bubble size indicates the number of DEGs in each pathway, and color represents the adjusted p-value (-log10).

**(b)** Summary of immune functional modules (Cytotoxicity, Activation, Migration, Cytokine/Chemokine, Inhibitory) for selected genes in NK92 and CD73-CAR-NK92 cells. Bubble size corresponds to the absolute log2 fold change, and color indicates the direction and magnitude of change (red, upregulated; blue, downregulated).

**(c-h)** Statistical quantification of the flow cytometry data presented in Figure 2g-l.

Bar graphs show mean fluorescence intensity (MFI) or percentage of positive cells for CD107a **(c)**, FASL **(d)**, NKG2D **(e)**, NKp46 **(f)**, TIGIT **(g)**, and NKG2A **(h)** on NK92 and CD73-CAR-NK92 cells following tumor co-culture.

**(i-k)** ELISA quantification of CCL3 **(i)**, CCL5 **(j)**, and CSF2 **(k)** levels in culture supernatants from NK92 and CD73-CAR-NK92 cells cultured alone or co-cultured with H1299 cells for 24 h.

**(l-o)** ELISA quantification of CCL3 **(l)**, CCL5 **(m)**, CSF1 **(n)**, and CSF2 **(o)** levels in culture supernatants from PBNK and CD73-CAR-PBNK cells cultured alone or co-cultured with H1299 cells for 24 h.

Data are presented as mean ± SD (n = 3 independent experiments). For **(c-h, i-o)**, statistical significance was determined using a two-way mixed-effects model followed by Šídák's multiple comparisons test.

**Supplementary Figure 3.**

Normal cell safety, adenosine resistance, 3D spheroid eradication, and antigen dependency validation.

**(a)** Cytotoxicity of NK92 and CD73-CAR-NK92 cells against normal bronchial epithelial BEAS-2B cells after 24 h of co-culture at the indicated effector-to-target (E:T) ratios.

**(b, c)** Representative time-course images of PC9 tumor spheroids (**b**) and mixed PC9/A549 spheroids (**c**) treated with PBS, NK92 cells, or CD73-CAR-NK92 cells. Scale bar, 200 μm.

**(d)** Extracellular adenosine levels measured by ELISA in culture supernatants following co-culture of NK92 or CD73-CAR-NK92 cells with LUAD cells at the indicated E:T ratios.

**(e)** qPCR analysis of CD73 mRNA expression in H1299 cells following transfection with three independent CD73-targeting siRNAs.

**(f, g)** Western blot analysis **(f)** and corresponding densitometric quantification **(g)** of CD73 protein expression in H1299 cells following siRNA-mediated knockdown.

**(h, i)** Western blot analysis **(h)** and corresponding densitometric quantification **(i)** of CD73 protein expression in A549 cells following lentiviral overexpression.

**(j)** Western blot analysis of PKA substrate phosphorylation in PBNK and CD73-CAR-PBNK cells following exposure to exogenous adenosine or co-culture with H1299 cells.

**(k)** Densitometric quantification of PKA substrate phosphorylation normalized to GAPDH.

**(l)** Real-time cytotoxicity of NK92 cells against H1299 cells over 24 h in the presence of exogenous adenosine, an A2AR antagonist, or their combination, with CD73-CAR-NK92 cells included for comparison.

**(m)** Real-time cytotoxicity of NK92 cells treated with an anti-CD73 antibody compared with untreated NK92 and CD73-CAR-NK92 cells over 24 h.

Quantitative data are presented as mean ± SD from three independent experiments unless otherwise indicated. Statistical significance was assessed using unpaired two-tailed Student’s *t*-tests for two-group comparisons and one-way or two-way ANOVA with appropriate multiple-comparison tests for multi-group comparisons, as indicated in the corresponding panels. *P* < 0.05 was considered statistically significant; ns, not significant.

**Supplementary Figure 4.**

3D spheroid assays with diverse NK sources.

**(a, b)** Representative bright-field images of H1299 **(a)** and PC9 **(b)** tumor spheroids treated with iNK cells or CAR-iNK cells.

**(c)** Representative images of H1299/A549 mixed spheroids treated with UBNK cells or CAR-UBNK cells, showing preferential elimination of CD73 ⁺ ⁺ H1299 cells.

**(d-f)** Representative images of A549 spheroids **(d)**, H1299 spheroids **(e)**, and H1299/A549 mixed spheroids **(f)** treated with PBNK cells or CAR-PBNK cells.

All scale bars, 200 μm. Images are representative of three independent experiments.

**Supplementary Figure 5.**

Intratumoral effector molecule levels and systemic toxicity assessment in the H1299 CDX model.

**(a-c)** ELISA quantification of intratumoral granzyme B **(a)**, TNF-α **(b)**, and IFN-γ **(c)** levels in tumor tissues harvested at the experimental endpoint from PBS-, NK92-, and CD73-CAR NK92-treated mice.

**(d)** Representative H&E staining images of heart, liver, spleen, lung, and kidney sections from NSG mice in the indicated treatment groups. Scale bar, 20 μm.

Data in **(a-c)** are presented as mean ± SD (n = 5-7 mice per group). Statistical significance was assessed by one-way ANOVA with Tukey's post hoc test.

**Supplementary Figure 6.**

Antitumor efficacy in the PC9 CDX model.

**(a)** Treatment schema of the PC9 subcutaneous CDX model.

**(b)** Longitudinal bioluminescence imaging of tumor burden in PBS, NK92, and CD73-CAR NK92 treated mice on days 0, 7, 14, 23, and 35 post-tumor inoculation.

**(c-e)** Individual tumor growth curves of mice in the PBS **(c)**, NK92 **(d)**, and CD73-CAR NK92 **(e)** groups.

**(f)** Kaplan-Meier survival curves of treated mice.

**(g)** Schematic illustration of peripheral blood and tumor tissue collection.

**(h-k)** Flow cytometric quantification of NK cell proportions in peripheral blood and tumor tissues at day 3 (**h, i**) and day 7 (**j, k**).

Data in **(c-f)** are presented as mean ± SD (n = 5-7 mice per group).

Statistical significance in **(a, d)** was determined using a two‑way ANOVA or mixed‑effects model was used, followed by Šídák's multiple comparisons test**.**

**Supplementary Figure 7.**

Endpoint analyses of NCG-PDX model.

**(a)** Representative images of excised tumors from NCG-PDX mice at the experimental endpoint in PBS, NK92, and CD73-CAR-NK92 treatment groups.

**(b)** Body weight curves of mice monitored throughout the treatment period.

**(c)** Intratumoral adenosine concentrations measured by ELISA in tumor homogenates from the indicated groups.

**(d)** Intratumoral GZMB levels measured by ELISA.

Data in **(b-d)** are presented as mean ± SD (n = 3 mice per group). Statistical significance in (c, d) was assessed by one-way ANOVA with Welch's correction (not assuming equal variances) followed by Dunnett's T3 multiple comparisons test.

**Supplementary Figure 8.**

Immune profiling of splenic populations and intratumoral effector molecules in the humanized PDX model.

**(a)** Representative flow cytometry gating strategy for splenic immune cells. Top row: FSC-SSC gate identifying the major splenic cell population. Middle row: DAPI⁻ gate excluding dead cells. Bottom row: CD45⁺ gate identifying human immune cells.

**(b-e)** Flow cytometric quantification of splenic NK cells **(b)**, dendritic cells **(c)**, CD8⁺ T cells **(d)**, and CD4^+^ T cells **(e)**.

**(f-i)** Proportions of activated cells among splenic NK cells (**f**, CD107a⁺), dendritic cells (**g**, CD80⁺), CD8⁺ T cells (**h**, CD69⁺), and CD4⁺ T cells (**i**, CD69⁺).

**(j)** Representative H&E staining images of spleen sections from healthy mice and tumor-bearing mice treated as indicated. Scale bar, 20 μm.

**(k-n)** ELISA quantification of CCL3 **(k),** CCL5 **(l)**, CSF1 **(m)**, and CSF2 **(n)** concentrations in blood and tumor homogenates from immune-humanized PDX mice.

**(o, p)** Flow cytometric quantification of the proportions of migrated dendritic cells **(o)** and T cells **(p)** in a Transwell migration assay using PBMCs and conditioned media from the NK92 system.

**(q, r)** Flow cytometric quantification of the proportions of migrated dendritic cells **(q)** and T cells **(r)** in a Transwell migration assay using PBMCs and conditioned media from the PBNK system.

Data in **(b-i, k-n)** are presented as mean ± SD (n = 3 mice per group). For **(b-i)**, statistical significance was assessed by one-way ANOVA test. For **(k-n)**, a two-way ANOVA or mixed-effects model was used. For **(o-r)**, data are presented as mean ± SD (n = 3 independent experiments), and statistical significance was determined using Brown-Forsythe and Welch ANOVA followed by Games-Howell multiple comparisons test.

**Supplementary Figure 9.**

Systemic safety and organ function assessment in the humanized PDX model.

**(a-e)** Serum levels of AST **(a)**, ALT **(b)**, creatinine **(c)**, uric acid **(d)**, and Fe **(e)** in healthy mice (n = 1, serving as a non-tumor baseline reference) and tumor-bearing mice treated with PBS (n = 3), NK92 cells (n = 3), or CD73-CAR NK92 cells (n = 3).

**(f, g)** Serum concentrations of IL-6 **(f)** and C-reactive protein **(g)** measured by ELISA in the indicated groups.

**(h)** Representative H&E staining images of heart, liver, spleen, lung, and kidney sections from healthy mice and tumor-bearing mice treated as indicated. Scale bar, 20 μm.

Data from tumor-bearing groups in **(a-g)** are presented as mean ± SD. Statistical comparisons among PBS, NK92, and CD73-CAR NK92 groups in **(a-g)** were performed by one-way ANOVA with Tukey's post hoc test. Due to the single-sample nature of the healthy group, no statistical comparisons were made against this baseline reference.

**Supplementary Table 1.**

Clinical characteristics of patients

| No. | Sex | Status | Stage | EGFR/ALK mutation status | Prior treatment | Sample Use |
| --- | --- | --- | --- | --- | --- | --- |
| P-01 | Female | Primary | IA1 | NA | NA | **IHC** |
| P-02 | Female | Primary | IA3 | EGFR | NA |  |
| P-03 | Female | Primary | IA1 | KRAS | NA |  |
| R-01 | Female | R/R | IA3 | NA | Postoperative |  |
| R-02 | Male | R/R | IB | NA | Postoperative |  |
| R-03 | Female | R/R | IA1 | EGFR | Postoperative |  |
| R-04 | Male | R/R | IA1 | NA | Postoperative |  |
| R-05 | Male | R/R | IA3 | NA | Postoperative |  |
| R-06 | Male | R/R | IA2 | EGFR | Postoperative |  |
| P-05 | Male | Primary | IA3 | EGFR | NA | **IF** |
| P-06 | Female | Primary | IA3 | NA | NA |  |
| P-07 | Female | Primary | IA3 | EGFR | NA |  |
| P-08 | Female | Primary | IA2 | EGFR | NA |  |
| R-09 | Male | R/R | IVA | NA | Postoperative |  |
| R-10 | Female | R/R | IIIB | NA | 3 cycles of chemoimmunotherapy |  |
| R-11 | Male | R/R | IIIB | NA | 4 cycles of chemoimmunotherapy |  |

| No. | Sex | Status | Stage | EGFR/ALK mutation status | Prior treatment | Sample Use |
| --- | --- | --- | --- | --- | --- | --- |
| P-04 | Male | Primary | IVA | ALK | NA | **PDX** |
| R-07 | Female | R/R | IIIB | NA | Post 3 cycles of chemoimmunotherapy, left upper lobectomy for lung adenocarcinoma, adjuvant targeted therapy |  |
| R-08 | Female | R/R | IIIB | EGFR | Almonertinib targeted therapy |  |
| R-12 | Female | R/R | IVA | EGFR | Almonertinib targeted therapy | **HuHSC-PDX** |
| R-13 | Female | R/R | IIIB | EGFR | Osimertinib followed by furmonertinib targeted therapy |  |
| R-14 | Female | R/R | IA1 | NA | Postoperative |  |

**Supplementary Table 2.**

Primers used for qPCR

| Gene | Forward | Reverse |
| --- | --- | --- |
| *CD73* | ATTCTAGAGCTAGCGAATTCATGTGTCCCCGAGCCGCG | CCGTCATGGTCTTTGTAGTCGGATCCCTATTGGTATAAAC |
| *h-GAPDH* | TGCACCACCAACTGCTTAGC | GGCATGGACTGTGGTCATGAG |

**Supplementary Table 3.**

**Antibodies**

| Primary antibodies | |  |  |  |
| --- | --- | --- | --- | --- |
| Antigen | **Species** | **Cat.No.** | **Company** | **Dilution** |
| *CD73* | Rabbit | ab317462 | abcam | 1:500(IF)  1:2000(WB) |
| *PKA* | Rabbit | 4782S | CST | 1:1000（WB） |
| *Phospho- PKA Substrate* | Rabbit | 9621S | CST | 1:1000（WB） |

| Second antibodies |  | | |
| --- | --- | --- | --- |
| Antigen | **Cat.No.** | **Company** | **Dilution** |
| HRP-conjugated Goat Anti-Rabbit IgG(H+L) | SA00001-2 | Proteintech | 1:5000(WB) |
| HRP-conjugated Goat Anti-Mouse IgG(H+L) | SA00001-1 | Proteintech | 1:5000(WB) |
| Donkey anti-Rabbit IgG Alexa Fluor 546 | A10040 | Invitroge™ | 1:500(IF) |

**Supplementary Table 4.**

FCM antibodies

| Antigen | Fluorochrome | Cat.No. | Company | Dilution |
| --- | --- | --- | --- | --- |
| *CD73* | FITC | 344015 | BioLegend | 1:200 |
| *CD56* | PE | 304606 | Biolegend | 1:200 |
| *CD107a* | APC/Cyanine7 | 328629 | Biolegend | 1:200 |
| *FasL* | APC | 306421 | Biolegend | 1:200 |
| *NKG2D* | PE/Cyanine7 | 320811 | Biolegend | 1:200 |
| *NKp46* | FITC | G3061 | Selleck | 1:200 |
| *TIGIT* | APC/Cyanine7 | 372734 | Biolegend | 1:200 |
| *NKG2A* | APC | 375108 | Biolegend | 1:200 |
| *CD45* | APC | H0093 | Selleck | 1:200 |
| *CD4* | PE/Cyanine7 | 344611 | BioLegend | 1:200 |
| *CD8* | FITC | 344703 | BioLegend | 1:200 |
| *CD69* | PE | 310906 | BioLegend | 1:200 |
| *CD80* | PE/Cyanine7 | L307.4 | Selleck | 1:200 |
| *CD3* | APC | 300312 | BioLegend | 1:200 |
| *HLA-DR* | APC | 307609 | BioLegend | 1:200 |

**Supplementary Table 5**

List of Elisa kits

| Reagent | Supplier | Cat. No. |
| --- | --- | --- |
| Human Adenosine ELISA Kit | Mlbio | ml063023 |
| Human Granzyme B (Gzms-B) ELISA Kit | Mlbio | ml060098 |
| Human Perforin 1 (PF1) ELISA Kit | Mlbio | ml105197 |
| High Sensitivity Human Interferon Gamma (IFN-γ) ELISA Kit | Elabscience | E-HSEL-H0007 |
| Human Tumor Necrosis Factor Alpha (TNF-α) ELISA Kit | Elabscience | E-EL-H0109 |
| Human Granulysin (GNLY) ELISA Kit | Elabscience | E-EL-H1618 |
| Human Macrophage Inflammatory Protein 1 Alpha (MIP-1α) ELISA Kit | Elabscience | E-EL-H6213 |
| Micro Human Regulated on Activation, Normal T Cell Expressed and Secreted (RANTES) ELISA Kit | Elabscience | E-MSEL-H0036 |
| Human Macrophage Colony-Stimulating Factor (M-CSF) ELISA Kit | Elabscience | E-EL-H0097 |
| Human Granulocyte-Macrophage Colony-Stimulating Factor (GM-CSF) ELISA Kit | Elabscience | E-EL-H0081 |

**Supplementary Table 6**

List of Abbreviations

| Abbreviation | Full Name |
| --- | --- |
| ACKR3 | Atypical Chemokine Receptor 3 |
| ALT | Alanine Aminotransferase |
| AST | Aspartate Aminotransferase |
| CAR | Chimeric Antigen Receptor |
| CCL3 | C‑C Motif Chemokine Ligand 3 |
| CCL4 | C‑C Motif Chemokine Ligand 4 |
| CCL5 | C‑C Motif Chemokine Ligand 5 |
| CDX | Cell line‑Derived Xenograft |
| CRP | C‑Reactive Protein |
| CRS | Cytokine Release Syndrome |
| CSF1 | Colony Stimulating Factor 1 |
| CSF2 | Colony Stimulating Factor 2 |
| CXCR3 | C‑X‑C Motif Chemokine Receptor 3 |
| CXCR6 | C‑X‑C Motif Chemokine Receptor 6 |
| SK | SK-MES-1 |
| DC | Dendritic Cell |
| ELISA | Enzyme‑Linked Immunosorbent Assay |
| EMT | Epithelial‑to‑Mesenchymal Transition |
| E:T | Effector‑to‑Target ratio |
| FASLG | Fas Ligand |
| GNLY | Granulysin |
| GvHD | Graft‑versus‑Host Disease |
| GZMA | Granzyme A |
| GZMB | Granzyme B |
| HLA‑DR | Human Leukocyte Antigen‑DR |
| ICANS | Immune Effector Cell‑Associated Neurotoxicity Syndrome |
| IFN‑γ | Interferon‑Gamma |
| iNK | iPSC‑Derived Natural Killer cell |
| iPSC | Induced Pluripotent Stem Cell |
| KEGG | Kyoto Encyclopedia of Genes and Genomes |
| LUAD | Lung Adenocarcinoma |
| MDSC | Myeloid‑Derived Suppressor Cell |
| NCG | NOD‑Prkdc^scid Il2rg^tm1 mice |
| NK | Natural Killer cell |
| NSCLC | Non‑Small Cell Lung Cancer |
| PBNK | Peripheral Blood‑Derived Natural Killer cell |
| PDX | Patient‑Derived Xenograft |
| PFS | Progression‑Free Survival |
| huHSC-PDX | Humanized hematopoietic stem cell patient-derived xenograft |
| PRF1 | Perforin‑1 |
| R/R | Relapsed/Refractory |
| scFv | Single‑Chain Variable Fragment |
| TIGIT | T‑cell Immunoreceptor with Ig and ITIM domains |
| TME | Tumor Microenvironment |
| TNF‑α | Tumor Necrosis Factor‑Alpha |
| Treg | Regulatory T cell |
| UBNK | Umbilical Cord Blood‑Derived Natural Killer cell |
